# Computational Study of Drugs Targeting Nuclear Receptors

**DOI:** 10.3390/molecules25071616

**Published:** 2020-04-01

**Authors:** Maša Kenda, Marija Sollner Dolenc

**Affiliations:** Faculty of Pharmacy; University of Ljubljana, Aškerčeva cesta 7, SI-1000 Ljubljana, Slovenia; masa.kenda@ffa.uni-lj.si

**Keywords:** endocrine-disrupting chemicals, drugs, databases, nuclear receptors, molecular docking, multidimensional QSAR.

## Abstract

Endocrine-disrupting chemicals have been shown to interfere with the endocrine system function at the level of hormone synthesis, transport, metabolism, binding, action, and elimination. They are associated with several health problems in humans: obesity, diabetes mellitus, infertility, impaired thyroid and neuroendocrine functions, neurodevelopmental problems, and cancer are among them. As drugs are chemicals humans can be frequently exposed to for longer periods of time, special emphasis should be put on their endocrine-disrupting potential. In this study, we conducted a screen of 1046 US-approved and marketed small-molecule drugs (molecular weight between 60 and 600) for estimating their endocrine-disrupting properties. Binding affinity to 12 nuclear receptors was assessed with a molecular-docking program, Endocrine Disruptome. We identified 130 drugs with a high binding affinity to a nuclear receptor that is not their pharmacological target. In a subset of drugs with predicted high binding affinities to a nuclear receptor with Endocrine Disruptome, the positive predictive value was 0.66 when evaluated with in silico results obtained with another molecular docking program, VirtualToxLab, and 0.32 when evaluated with in vitro results from the Tox21 database. Computational screening was proven useful in prioritizing drugs for in vitro testing. We suggest that the novel interactions of drugs with nuclear receptors predicted here are further investigated.

## 1. Introduction

Endocrine-disrupting chemicals (EDCs) are a subject of an increasing concern in our society. Exposure to them has been lined with obesity, diabetes mellitus, infertility, impaired thyroid and neuroendocrine functions, neurodevelopmental problems, and cancer [1]. The United States Environmental Protection Agency (USEPA) defines an endocrine-disrupting chemical (EDC) as “an exogenous agent that interferes with the production, release, transport, metabolism, binding, action, or elimination of natural hormones in the body responsible for the maintenance of homeostasis and the regulation of developmental processes” [2], while the World Health Organization (WHO) defines it as “an exogenous substance or mixture that alters function(s) of the endocrine system and consequently causes adverse effects in an intact organism, or its progeny, or (sub)populations” [3]. In 2016, the European Commission proposed an EDC definition to include known adverse effects “in an intact organism, or its progeny, or (sub)populations” [4]. The proposed change would mean some chemicals would not withstand the novel definition but would be classified as EDCs by the current one. Moreover, proposed criteria included a requirement to show EDC’s health effects on human data, the obtaining of which is lengthier and more expensive than obtaining data with currently used alternative methods [4]. The request for human data would make it merely impossible to define any novel chemical as an EDC. Though the proposed criteria were not implemented, the need to develop better alternative methods for the identification of EDCs remains.

The most well-known mechanism of action of EDCs is their ability to act as agonists and antagonists of nuclear hormone receptors. Upon binding of a ligand to a nuclear receptor, the receptor homo- or heterodimerizes and is translocated in the cell nucleus where it acts as a transcription factor regulating a vast number of genes and eliciting numerous physiological responses. Among such receptors are androgen receptor (AR); estrogen receptors α (ERα) and β (ERβ); glucocorticoid receptor (GR); liver X receptors α (LXRα) and β (LXRβ); peroxisome proliferator-activated receptors α (PPARα), β (PPARβ), and γ (PPARγ); retinoid X receptor α (RXRα); and thyroid receptors α (TRα) and β (TRβ). Activation of those receptors regulates processes important for reproductive and developmental health, behavior, and the immune system [1]. The adverse outcome pathway (AOP) is a conceptual framework used in toxicological risk assessment. It is a sequence of events in a biological system that leads to an adverse outcome. The adverse outcome pathway starts with a molecular initiating event, defined as “the initial interaction between a molecule and a biomolecule or biosystem that can be causally linked to an outcome via a pathway”, and is followed by several downstream key events, causing an adverse outcome [5]. Binding to a nuclear receptor is a molecular initiating event in several AOPs developed by the Organisation for Economic Co-operation and Development (OECD), e.g., “The AOP on Upregulation of Thyroid Hormone Catabolism via Activation of Hepatic Nuclear Receptors, and Subsequent Adverse Neurodevelopmental Outcomes in Mammals”, “The AOPs Linking Aromatase Inhibition, Androgen Receptor Agonism, Estrogen Receptor Antagonism, or Steroidogenesis Inhibition, to Impaired Reproduction in Small Repeat-Spawning Fish Species”, and “The AOP on CAR and PPARα-mediated pathways to non-genotoxic rodent liver cancer” [6]. Ligand binding of an EDC to a nuclear receptor could be considered as a molecular initiating event for many endocrine-related adverse outcomes in the future.

As drugs are chemicals we are frequently exposed to on a daily basis, in some cases for longer periods (up to a lifetime), and in higher quantities compared to other groups of EDCs (e.g., flame retardants, cosmetic ingredients, and plasticizers), special emphasis should be put on their endocrine-disrupting potential. In addition to known and well-documented exposure, pharmacologically active substances and/or their metabolites are also present in the environment (e.g., surface and drinking water and soil) and can enter the food chain (e.g., via meat produce, fish, and crops) [7,8,9]. In 2018, the European Medicines Agency issued a draft of revised guidelines on the environmental risk assessment of medicinal products for human use, which requires data on the effects on development and reproduction (including data on interacting or interfering with endocrine receptors) for all human drugs submitted for marketing authorization [10].

In this study, we set out to screen US-approved and marketed small-molecule drugs for endocrine-disrupting potential, using docking to nuclear receptors with Endocrine Disruptome (ED)—a program developed by our research group. Docking to nuclear receptors with VirtualToxLab (VTL) and data on the in vitro activity of drugs from in the Tox21 database will be used for assessing the positive predictive value (PPV) of ED.

## 2. Results

Molecular docking with ED was used to predict the binding of US-approved and marketed small-molecule drugs from the NCATS (National Center for Advancing Translational Sciences) Inxight: Drugs portal (version 1.1) to nuclear receptors [11,12]. Due to limitations of the ED (described in Section 4. Materials and Methods), 3.6% of the initial database entries were omitted, and a total of 1046 drugs were considered. Results where strong binding to a nuclear receptor was predicted were further evaluated by another in silico docking tool, VTL, and with in vitro data on reporter cell lines from the Tox21 database [13,14,15,16,17].

### 2.1. Screening of US-Approved and Marketed Drugs with ED

#### 2.1.1. Binding Affinity Distribution Across Nuclear Receptors

**Figure 1:**
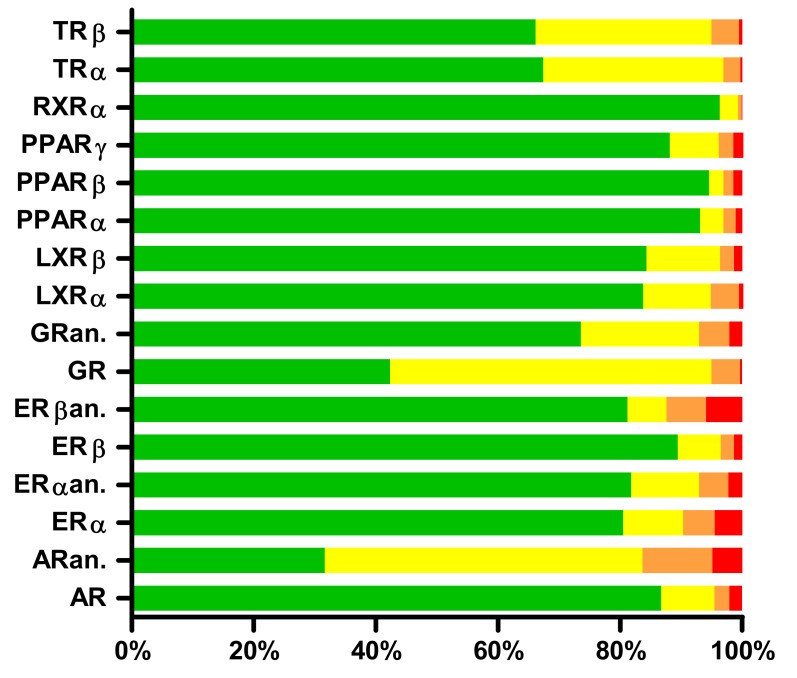
Binding affinity distribution of 1046 tested drugs for 12 nuclear receptors as assayed with Endocrine Disruptome. Green corresponds to a low probability of binding, yellow and orange correspond to a medium probability of binding, and red corresponds to a high probability of binding [12]. AR—agonist conformation of androgen receptor; ARan—antagonist conformation of androgen receptor; ERα—agonist conformation of estrogen receptor α; ERαan—antagonist conformation of estrogen receptor α; ERβ—agonist conformation of estrogen receptor β; ERβ—antagonist conformation of estrogen receptor β; GR—agonist conformation of glucocorticoid receptor; GR—antagonist conformation of glucocorticoid receptor; LXRα—liver X receptor α; LXRβ—liver X receptor β; PPARα—peroxisome proliferator-activated receptor α; PPARβ—peroxisome proliferator-activated receptor β; PPARγ—peroxisome proliferator-activated receptor γ; RXRα—retinoid X receptor α; TRα—thyroid receptor α; TRβ—thyroid receptor β.

The results of the predicted binding affinity distribution with ED for 1046 across 12 nuclear receptors are shown in Figure 1. Results are color-coded; green corresponds to a low probability of binding, yellow and orange correspond to a medium probability of binding, and red corresponds to a high probability of binding. Out of 1046 drugs tested, 22 exerted strong binding for the agonist conformation of AR, 51 for the antagonist conformation of AR, 47 for the agonist conformation of ERα, 24 for the antagonist conformation of ERα, 14 for the agonist conformation of ERβ, 62 for the antagonist conformation of ERβ, 4 for the agonist conformation of GR, 22 for the antagonist conformation of GR, 6 for LXRα, 14 for LXRβ, 11 for PPARα, 15 for PPARβ, 15 for PPARγ, 1 for RXRα, 3 for TRα, and 6 for TRβ. The most frequently bound receptors are therefore ERβ with 7.3% of the predicted strong binders among the screened drugs, followed by AR with 7.0% of the predicted strong binders among the screened drugs, and ERα with 6.8% of the predicted strong binders among the screened drugs. A complete list of 1046 drugs tested in this study with results for each receptor is available in Table S1: US-approved and marketed drugs with Endocrine Disruptome (ED)-predicted binding to respective nuclear receptors.

#### 2.1.2. Identification of Drugs with Strong Binding to Nuclear Receptors

In the next step, we removed drugs that work pharmacologically by binding to a nuclear receptor (i.e., drugs of Anatomical Therapeutic Chemical (ATC) Classification System groups “D07 Corticosteroids, dermatological preparations”; “L02 Endocrine therapy”; “G02 Other gynecologicals”; “G03 Genito—urinary system and sex hormones”; and retinoids from group “D Dermatologicals” were excluded). The remaining strong binders (130) and the receptors they bind to are listed in Table 1. For simplicity, we will further refer to this list as “prioritized drugs”.

**Figure 2:**
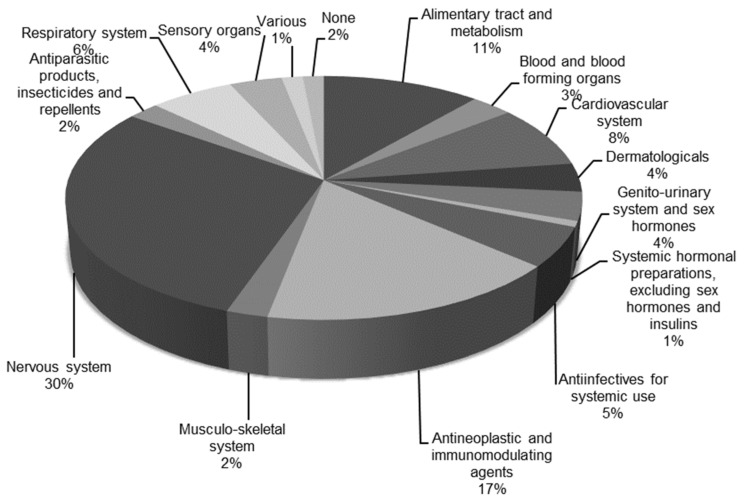
Distribution of prioritized drugs across the Anatomical Therapeutic Chemical (ATC) Classification System groups. Drugs that bind to a nuclear receptor as their mode of action were excluded. Total number of drugs represented in this figure is 130.

Prioritized drugs are represented in all ATC groups (Figure 2). The most represented groups are group “N, nervous system” with 39 drugs and group “L, antineoplastic and immunomodulating agents” with 22 drugs, followed by group “A, alimentary tract and metabolism” with 15 drugs and group “C, cardiovascular system” with 11 drugs. Other ATC groups have fewer than 10 drugs.

**Figure 3:**
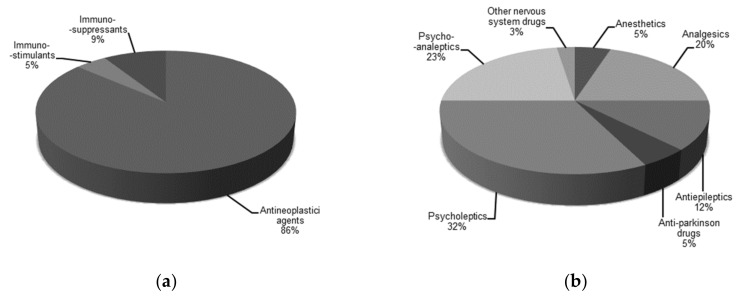
Distribution of prioritized drugs within the Anatomical Therapeutic Chemical (ATC) Classification System groups: (**a**) antineoplastic and immunomodulating agents and (**b**) the nervous system. Drugs that bind to a nuclear receptor as their mode of action were excluded. Total number of drugs represented in this figure is 22 in (**a**) and 39 in (**b**).

Further dissection of drugs in groups “L, antineoplastic and immunomodulating agents” and “N, nervous system” is presented in Figure 3. Group “L, antineoplastic and immunomodulating agents” consists of 19 antineoplastic agents—out of which, 14 are tyrosine kinase inhibitors, with two immunosuppressants and an immunostimulant (Figure 3a). Group “N, nervous system” consists of 13 psycholeptics, 9 psychoanaleptics, 8 analgesics, 5 antiepileptics, 2 anti-Parkinson drugs, 2 anesthetics, and 1 other nervous system drug (Figure 3b). The most well-represented group among the psychoanaleptics are antidepressants—four of which are nonselective monoamine reuptake inhibitors, three are selective serotonin reuptake inhibitors, and two are other antidepressants. Additionally, there are seven benzodiazepine derivatives among the psycholeptics, belonging to subcategories of anxiolytics or hypnotics and sedatives.

### 2.2. Positive Predictive Value of ED Prediction

Positive results for prioritized drugs were further evaluated with another docking in, silico tool, VTL, and with the in vitro results on reporter cell lines from the Tox21 database.

#### 2.2.1. Evaluation with VTL

A subset of prioritized drugs was tested with VTL. The positive predictive value was calculated (Equation (1)):

PPV = TP / (TP + FP),(1)

where TP and FP represent true positives and false positives, respectively. A drug was considered a TP if the ED predicted strong binding to a nuclear receptor and binding to that receptor was predicted with VTL. A drug was considered a FP if the ED predicted strong binding to a nuclear receptor and VTL predicted “not binding” for that receptor.

The positive predictive value for prioritized drugs was 0.66 when evaluated with VTL.

#### 2.2.2. Evaluation with Tox21 Database

Further, a subset of prioritized drugs was compared to in vitro results on reporter cell lines obtained from the Tox21 database, if available. A total of 53 drug-receptor interactions for prioritized drugs were found in the Tox21 database, and a PPV of 0.32 was calculated (Equation (1)). A drug was considered a TP if the ED predicted strong binding to a nuclear receptor and the drug exerted activity in cell assays for that receptor. A drug was considered a FP if the ED predicted strong binding to a nuclear receptor and the drug exerted no activity in cell assays for that receptor.

### 2.3. Cell-Based In Vitro Assays

**Figure 4:**
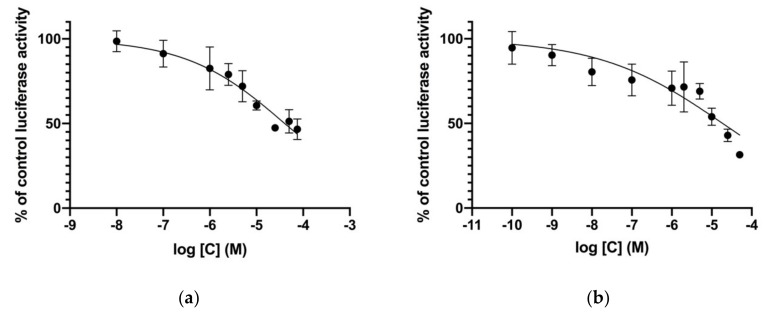
Dose-response curves for Endocrine Disruptome (ED)-predicted antagonist activities in luciferase reporter cell lines for: (**a**) irinotecan on GR in the MDA-kb2 cell line and (**b**) palonosetron on AR in the AR-EcoScreen cell line. Mean and SD of at least three independent experiments are shown.

Two drugs, irinotecan and palonosetron, were tested in luciferase reporter cell lines for activity on GR in the MDA-kb2 cell line and for activity on AR in the AR-EcoScreen cell line. Endocrine Disruptome-predicted antagonism on GR was confirmed for irinotecan (IC_50_ = 40.32 μM; dose-response curve is shown in Figure 4a). For palonosetron, a modulation of AR was confirmed. Palonosetron is an antagonist on AR (IC_50_ = 18.65 μM; dose-response curve is shown in Figure 4b), while it was tested negative at the highest noncytotoxic concentration of 10 μM in an agonist assay in the AR-EcoScreen cell line (data not shown).

## 3. Discussion

Both in silico tools used here are listed among the software tools for the in silico prediction of binding to nuclear receptors in guidance for the identification of endocrine disruptors by the European Chemical Agency and the European Food Safety Authority [18]. The guidance emphasizes the applicability of in silico predictions in providing information on molecular initiating events and making informed decisions on further testing strategies. The importance of considering the performance and applicability domain of in silico tools is stressed.

The ED tool was developed for the toxicological screening of new compounds; therefore, it was designed in a way to predict negative results with greater accuracy, as opposed to correctly predicting positive results [12]. Consequently, the program predicts fewer false negatives (FN), but there are more FP. Fewer FN assure a better safety assessment of new chemicals. Here, we used ED to find possible endocrine disruptors among the drugs. As positive results were searched for, we expected a low PPV (the ratio of true positives and all predicted positive results). Hence, validations of predicted positive results with another in silico tool, VTL, and in vitro results in the Tox21 database were performed.

Advantages of the ED as compared to VTL are that the ED is freely available via a web interface, the compound can easily be submitted by a simple SMILES input, and the calculation is quick. Limitations of the program in terms of the features of compounds that cannot be submitted to the ED are described in the Methods section. Conversely, VTL is not as easy to use, but it has an obvious and very valuable advantage over the ED—in addition to molecular modeling of the receptor interactions, VTL uses multidimensional QSAR modeling of the receptor-based activity, which yields quantitative binding information, i.e., a concentration at which an interaction with a nuclear receptor is predicted.

In addition to the ED having fewer false negatives at the cost of the PPV, as described above, the calculation of the PPV in this study has some limitations. VirtualToxLab is more advanced than the ED in terms of the computational background of both interfaces. We assumed VTL results as TP and TN, as it is known that false-positive results in the ED are yielded due to the program’s preference to correctly predict negative results, while false-positive results in VTL are attributed only to pharmacokinetic effects [14]. Though, VTL is not necessarily more accurate than the ED, as false-negative results are known to emerge for very small compounds. To some extent, this was avoided by database preparation with a restriction on molecular weight, as described in the Methods section. Additionally, VTL does not discriminate against the agonist and antagonist conformations of receptors, and the interpretation of those results might be intricate. To exemplify, in the case of palonosetron ED-predicted binding to AR for both agonist and antagonist receptor conformations, while VTL predicted no binding to AR. Our in vitro experiments confirmed AR antagonism for palonosetron with IC_50_ of 18.65 μM, but ED-predicted AR agonism was not confirmed at the highest noncytotoxic concentration. Therefore, the PPV for the ED of 0.66, calculated from an evaluation with VTL, might, in fact, be higher.

A major limitation of the evaluation with in vitro results in the Tox21 database is the possible cytotoxicity of the compounds. Though activity yielding due to cytotoxicity in antagonist-type assays was excluded from consideration, cytotoxicity might have caused some agonists to be interpreted as not active. Tox21 assays do not include OECD-validated cell lines AR-EcoScreen and hERalpha-HeLa-9903 for the identification of compounds with activity on AR and ERα, respectively [19,20]. Additionally, active concentrations of a compound might be between two tested concentrations in Tox21 assays, i.e., between an inactive and a cytotoxic concentration. Therefore, a positive result would remain unnoticed. An example of this phenomenon might be a Tox21 result on the estrogenic activity of paroxetine—Tox21 high-throughput assays for the activity of paroxetine on the estrogen receptor, namely, assays tox21-er-bla-agonist-p2, tox21-er-bla-antagonist-p1, tox21-er-luc-bg1-4e2-agonist-p2, tox21-er-luc-bg1-4e2-antagonist-p1, and tox21-er-luc-bg1-4e2-antagonist-p2, did not identify any activity of paroxetine on the ER, while the agonist activity of 10 and 20-μM paroxetine was shown in two ER-positive cell lines, MCF-7 and T47D, in a 96-well format [21]. Similarly, no antagonist activity of irinotecan on the GR at concentrations tested in the Tox21 assay tox21-gr-hela-bla-antagonist-p1 was seen, but we confirmed GR antagonism in the MDA-kb2 cell line with an IC_50_ of 18.65 μM. The difference in the outcomes is, hence, most likely due to more micromolar concentrations tested in our in vitro experiment. Another limitation of all cell-based assays is passing through the cell membrane. This could cause a lower concentration of a chemical at the nuclear receptor and result in a FP in our evaluation with in vitro results from the Tox21 database. The number of FP in the PPV calculated from an evaluation with Tox21 might really be lower due to the shortcomings of Tox21 assays, and a PPV higher than 0.32 might be expected. On the other hand, compounds might inhibit the luciferase reporter gene used in Tox21 assays and produce false-positive results in antagonism-type cell-based assays or stabilize the luciferase reporter gene and produce false-positive results in agonism-type cell-based assays [22,23].

Another level of complexity in assessing the endocrine-disrupting potential of drugs is added when the question of toxicological relevance for a compound with endocrine activity at concentrations much higher than plasma concentrations is addressed. This might be important for evaluating in silico results, such as in this study, but might not be of concern biologically. On the other hand, the inactivity of a compound at plasma concentrations in short-term exposure studies (e.g., in screening assays in the Tox21 program) is not an indication that the compound does not lead to adverse effects long-term. Hence, if the mechanism of action of EDCs, i.e., binding to nuclear receptors, is predicted or seen in vitro, such compounds should undergo further evaluation of long-term endocrine adverse effects. This is especially important for drugs where exposure is higher than with other groups of EDCs. Based on our results and a certain extent of structure similarity in ATC groups, special emphasis should be put on novel tyrosine kinase inhibitors, antidepressants, and benzodiazepine-type anxiolytics or hypnotics and sedatives. Regarding the development of alternative methods for the identification of EDCs among drugs, efforts should be made to better evaluate AR-, ERα-, and ERβ-mediated adverse effects, as these nuclear receptors were most frequently affected in this study.

## 4. Materials and Methods

### 4.1. Database Preparation

The NIH (National Institutes of Health) NCATS Inxight: Drugs portal (version 1.1, NCATS, Bethesda, MD, USA) was used to create a database of marketed US-approved drugs, freely available at https://drugs.ncats.io/ [11]. Both over-the counter and prescription drugs were considered. The portal encompasses data on US-approved drugs from the following sources: DailyMed, Drugs@FDA, OrangeBook, and Code of Federal Regulations Title 21—Over-the-Counter Drugs. Only entries with “chemical” and “substance Class” were used, while proteins, mixtures, polymers, nucleic acids, and structurally diverse drugs were filtered out. Additionally, due to the prerequisites of computational tools ED and VTL, compounds with molar masses below 60 and above 600 g/mol were removed from the database.

### 4.2. Docking with ED

The ED docking program (National Institute of Chemistry, Ljubljana, Slovenia) was used to estimate the binding affinities of drugs to 12 nuclear receptors: AR, ERα, ERβ, GR, LXRα, LXRβ, PPARα, PPARβ, PPARγ, RXRα, TRα, and TRβ. For four receptors (AR, ERα, ERβ, and GR), both agonist and antagonist conformations were considered. For the docking simulation, Docking Interface for Target Systems (DoTS) was used, while the docking calculation for drugs docked in a receptor’s ligand binding domain was done with AutoDock Vina. Receptor structures in the ED were validated with a database of active compounds, a database of compounds that have an agonist mode of action, and of a database that has an antagonist mode of action for each receptor. Based on the area-under-curve (AUC) evaluation of the receiver operating characteristic (ROC) curve (where sensitivity is plotted against 1 minus specificity), three threshold values of binding free energies were determined for each receptor. Thresholds translate to color-coded output results of the program: green corresponds to a low probability of binding (sensitivity > 0.75), yellow (0.5 < sensitivity < 0.75) and orange (0.25 < sensitivity < 0.5) correspond to a medium probability of binding, and red (sensitivity < 0.25) corresponds to a high probability of binding. Limitations for the compounds submitted to the ED are: molecular weight over 600 g/mol, multiple ionization of a compound, and boron-containing compounds. The program is a freely accessible web interface at http://endocrinedisruptome.ki.si/, and receptor structures prepared for docking are available for download as pdbqt files [12].

### 4.3. Docking with VTL

VirtualToxLab platform (version 5.8, Biographics Laboratory 3R, Basel, Switzerland) for estimating the toxic potential of compounds was used to predict the interactions of drugs with nuclear receptors for a subset of drugs with ED-predicted strong binding to a nuclear receptor and that do not target a nuclear receptor as a pharmacological mode of action. An intersection of receptors available in both the ED and VTL was considered: AR, ERα, ERβ, GR, LXRα, PPARγ, TRα, and TRβ. For an ED-predicted drug-receptor interaction in the subset of the drugs, the interaction with the respective receptor was assayed with VTL. The program evaluates the binding affinity by automated, flexible docking with Yeti/AutoDock. This provides for the assessment of all orientations and conformations of small molecules in the binding site. Additionally, VTL uses a multidimensional QSAR process using the mQSAR software (Quasar), which considers orientation, position, different solvation, protonation, conformation, induced-fit models, and the tautomeric state of the small molecules. As a result, the data are provided as concentrations at which the compounds are predicted to interact with a nuclear receptor. If no interaction is predicted or the concentration of interaction would be higher than 100 μM, the program returns “not binding” as a result. The program was developed by Biographics Laboratory 3R, and its license is freely accessible for universities and nonprofit organizations, as well as governmental agencies, at http://www.biograf.ch/ [13,14].

### 4.4. Tox21 Dataset Retrieval

The Toxicology in the 21st Century (Tox21) program aims at the developing and evaluating of high-throughput screening methods for toxicological risk assessment. It is a product of collaboration of the EPA, National Toxicology Program (NTP), NCATS, and the Food and Drug Administration (FDA). Tox21 data on screening of more than 10,000 compounds in cell-based assays are publicly available. Activities of several transcription factors in gene reporter assays were tested upon exposure to compounds in the Tox21 database, assays for the activation of some endocrine nuclear receptors among them. An intersection of receptors available in both the ED and Tox21 database was considered: AR, ERα, GR, RXRα, PPARγ, and TR. Tox21 data visualization tool Tox21 activity profiler (beta version, National Institute of Environmental Health Sciences, Durham, NC, USA) was used to access the data available at https://sandbox.ntp.niehs.nih.gov/tox21-activity-browser/ [15,16,17]. A list of CAS numbers of the subset of prioritized drugs was submitted to the tool. Activity due to cytotoxicity for antagonist-type calls was excluded. For an ED-predicted drug-receptor interaction in the subset of the drugs, interactions with the respective receptors were considered positive for AR if activity was seen in any of the following assays: tox21-ar-bla-agonist-p1, tox21-ar-bla-antagonist-p1, tox21-ar-mda-kb2-luc-agonist-p1, tox21-ar-mda-kb2-luc-antagonist-p1, or tox21-ar-mda-kb2-luc-antagonist-p2; for ERα, if activity was seen in any of the following assays: tox21-er-bla-agonist-p2, tox21-er-bla-antagonist-p1, tox21-er-luc-bg1-4e2-agonist-p2, tox21-er-luc-bg1-4e2-antagonist-p1, or tox21-er-luc-bg1-4e2-antagonist-p2; for GR, if activity was seen in any of the following assays: tox21-gr-hela-bla-agonist-p1 or tox21-gr-hela-bla-antagonist-p1; for RXRα, if activity was seen in the tox21-rxr-bla-agonist-p1 assay; for PPARγ, if activity was seen in the tox21-pparg-bla-agonist-p1 or tox21-pparg-bla-antagonist-p1 assays; and for TR, if activity was seen in the tox21-gh3-tre-agonist-p1 or tox21-gh3-tre-antagonist-p1 assays. Inconclusive results (where activity is at a concentration higher than 100 μM) were deemed as negative results.

### 4.5. Cell-Based Assays

The MDA-kb2 cell line (purchased from the American Type Culture Collection, Manassas; VA, USA, refence number CRL-2713) and AR-EcoScreen cell line (purchased from the Japanese Collection of Research Bioresources Cell Bank, Ibaraki, Japan, reference number JCRB1328) were used for cell-based assays. The MDA-kb2 cell line was derived from the MDA-MB-453 human breast cancer cell line by stable transfection with the firefly luciferase gene controlled by a glucocorticoid-inducible promoter [24]. The MDA-kb2 were maintained in Leibovitz’s L-15 medium (Sigma-Aldrich, Saint Louis, MO, USA), supplemented with 10% dextran-charcoal-stripped fetal bovine serum (Gibco, Thermo Fisher Scientific, Waltham, MA, USA), 100 U/mL penicillin (Sigma-Aldrich), and 100 µg/mL streptomycin (Sigma-Aldrich). Antagonist assay with MDA-kb2 was performed as previously described by Kolšek et al. [25]. AR-EcoScreen is an OECD-validated cell line that was derived from the Chinese hamster ovary (CHO-K1) cell line by stable transfection with a human androgen receptor, firefly luciferase gene controlled by an androgen-inducible promoter, and a constitutively expressed sea pansy luciferase gene. AR-EcoScreen were maintained in phenol-red-free Dulbecco’s modified Eagle’s medium (DMEM)/F-12 (Gibco), supplemented with 5% fetal bovine serum (Gibco), 200 µg/mL zeocin (InvivoGen, Toulouse, France), 100 µg/mL hygromycin (Sigma-Aldrich), 100 U/mL penicillin (Sigma-Aldrich), and 100 µg/mL streptomycin (Sigma-Aldrich). All experiments with AR-EcoScreen were performed according to the OECD test guideline 458 [20]. Irinotecan (CAS 100286-90-6) and palonosetron (CAS 135729-61-2) were purchased from Alfa Aesar (Ward Hill, MA, USA) and Tokyo Chemical Industry (Tokyo, Japan), respectively. Hydrocortisone (CAS 50-23-7), dihydrotestosterone (CAS 521-18-6), and DMSO (CAS 67-68-5) were purchased from Sigma-Aldrich.

## 5. Conclusions

While assessing if a compound fulfills the WHO criteria for EDCs and “alters function(s) of the endocrine system and consequently causes adverse effects” is a very complex task; evaluating a molecular interaction between a compound and a nuclear receptor is not [3]. Hence, we expect binding to nuclear receptors will often be defined as a molecular initiating event in AOPs yet to be developed for EDCs and, consequently, used in toxicological screening assays of compounds.

In light of the novel European Medicines Agency draft guidelines that require data on the endocrine activity of drugs submitted for approval, we propose that already-approved drugs are assessed in the same manner. Based on the computational evaluation done here, we suggest prioritized drugs for such assessments.

## Figures and Tables

**Table 1:** Medicines with Endocrine Disruptome-predicted strong binding to respective nuclear receptors.

Name ^1^	Nuclear Receptor ^2^	Binding Free Energy (kcal/mol)
Amitriptyline	ERβ	−9.2_(an)_
Amoxicillin	AR	−8.5_(an)_
Apixaban	PPARγ	−10.5
Aprepitant	GR; LXRβ; PPARγ	−10.0_(an)_; −12.4;
Artemether	AR; ERβ	−8.7_(an)_; −9.6
Asenapine	ERα	−9.6
Atovaquone	GR; LXRα; LXRβ; TRβ	−11.0; −12.2; −12.4; −10.8
Axitinib	GR	−10.8
Azilsartan	ERβ; GR	−9.8_(an)_; −9.9_(an)_
Belinostat	TRα	−10.5
Benzatropine	ERα; ERβ	−9.6; −9.6_(an)_
Brexpiprazole	ERα	−9.4
Butenafine	ERβ	−9.6_(an)_
Butorphanol	ERα	−9.3
Cabozantinib	ERβ; GR	−9.1_(an)_; −10.2_(an)_
Candesartan	ERβ	−9.6_(an)_
Carbamazepine	AR; ERα; ERβ	−8.8_(an)_; −9.6; −9.8_(an)_
Celecoxib	ERα; ERβ	−9.6 and −9.9_(an)_; −9.3
Ceritinib	ERβ	−9.2_(an)_
Cinacalcet	AR; ERα; ERβ; TRα	−8.5_(an)_; −10.0; −9.7 and −9.2_(an)_; −10.3
Clonazepam	ERβ	−9.4_(an)_
Clorazepate	ERα	−9.4
Cocaine	AR	−8.4_(an)_
Codeine	AR	−9.1 and −8.7_(an)_
Conivaptan	ERβ; GR	−9.5; −10.9 and −11.1_(an)_
Cyclobenzaprine	AR; ERα; ERβ	−8.6_(an)_; −9.9; −9.6_(an)_
Cyproheptadine	ERα; ERβ	−10.2; −9.3_(an)_
Dabrafenib	ERβ	−9.7_(an)_
Dacomitinib	GR	−9.6_(an)_
Darifenacin	ERα	−9.7
Deacetylbisacodyl	ERα; ERβ	−9.5; −9.4_(an)_
Deferasirox	ERα	−9.4_(an)_
Diazepam	ERα	−9.5
Dihydroergotamine	ERα; ERβ; GR	−11.6_(an)_; −10.6_(an)_; −11.6_(an)_
Duvelisib	ERβ; LXRα; LXRβ	−9.2_(an)_; −12.5; −12.5
Efinaconazole	AR	−8.9_(an)_
Eltrombopag	ERα; LXRα; LXRβ	−9.9_(an)_; −12.4; −12.3
Entecavir	AR	−8.5
Ergotamine	GR	−11.3_(an)_
Eslicarbazepine	ERα	−9.9
Estazolam	ERβ	−9.1_(an)_
Ezetimibe	ERα; ERβ; PPARγ	−10.5 and −9.8_(an)_; −9.7_(an)_; −10.7
Fenoldopam	AR	−8.9_(an)_
Fentanyl	AR	−8.8_(an)_
Fexofenadine	ERα	−9.4
Flavoxate	ERα	−9.6
Flumazenil	AR	−8.5_(an)_
Fluorescein	ERβ	−9.7_(an)_
Fluoxetine	AR	−8.5_(an)_
Glimepiride	ERα	−10.0
Glipizide	ERα	−9.4 and −9.4_(an)_
Glyburide/glibenclamide	ERα	−9.4
Granisetron	AR	−8.8_(an)_
Hydrocodone	AR	−8.7_(an)_
Hydromorphone	AR; ERα; ERβ	−9.2_(an)_; −9.8; −9.4 and −9.5_(an)_
Ibrutinib	GR; LXRβ; PPARγ	−9.9_(an)_; −12.7; −10.5
Imatinib	ERα	−9.8_(an)_
Indapamide	ERα	−9.5_(an)_
Irbesartan	ERα; ERβ	−10.0_(an)_; −9.4_(an)_
Irinotecan	ERα; ERβ; GR	−9.4_(an)_; −9.7_(an)_; −11.1_(an)_
Ketoconazole	ERα; ERβ	−9.5 and −9.9_(an)_; −9.3_(an)_
Ketorolac	AR	−8.5_(an)_
Lapatinib	PPARγ	−11.0
Larotrectinib	ERβ; LXRβ	−9.1_(an)_; −12.2
Levorphanol	AR; ERα; ERβ	−9.1 and −8.9_(an)_; −9.5; −9.5 and −9.1_(an)_
Lorazepam	ERβ	−9.6
Lumacaftor	ERβ; GR; LXRβ	−9.9_(an)_; −10.3_(an)_; −12.7
Lurasidone	ERβ; GR; LXRα; LXRβ	−9.2_(an)_; −10.0_(an)_; −12.1; −12.6
Maprotiline	ERα	−9.6
Maraviroc	ERα; ERβ	−9.4_(an)_; −9.7_(an)_
Meclizine	ERβ	−9.3_(an)_
Meclofenamic acid	AR	−8.5_(an)_
Mefloquine	AR; ERβ; TRα	−9.8_(an)_; −9.4_(an)_; −10.5
Methscopolamine	AR	−8.5_(an)_
Methylnaltrexone	TRβ	−10.6
Montelukast	ERα; ERβ; PPARγ	−10.2_(an)_; −9.5_(an)_; −10.4
Morphine	ERα; ERβ	−9.4; −9.5
Naftifine	AR; ERβ	−8.7_(an)_; −9.5
Naldemedine	ERβ; GR	−9.2_(an)_; −10.8_(an)_
Naltrexone	AR	−8.7_(an)_
Nebivolol	AR; ERα	−8.7_(an)_; −9.5 and −9.5_(an)_
Nelfinavir	ERα; ERβ; GR; PPARγ	−10.5_(an)_; −9.6_(an)_; −10.9_(an)_; −11.5
Netupitant	GR	−10.8_(an)_
Nintedanib	GR	−10.0_(an)_
Nitisinone	AR	−8.9_(an)_
Nortriptyline	ERβ	−9.1_(an)_
Olaparib	GR; LXRβ	−10.7_(an)_; −12.9
Olopatadine	AR; ERα	−8.7_(an)_; −9.5
Oxazepam	ERβ	−9.4
Oxcarbazepine	ERα	−9.9
Oxymorphone	AR; ERβ	−9.0_(an)_; −9.4 and −9.2_(an)_
Palbociclib	GR	−9.9_(an)_
Paliperidone	ERα	−9.6_(an)_
Palonosetron	AR; ERα	−10.0 and −9.3_(an)_; −9.6
Paroxetine	ERα	−9.5
Penicillin g/benzylpenicillin	AR	−8.5_(an)_
Perampanel	ERβ	−9.6_(an)_
Phenindamine	ERα; ERβ	−9.6; −9.5 and −9.7_(an)_
Pimozide	ERα; ERβ; GR; LXRα; LXRβ; PPARγ	−9.9; −9.9_(an)_; −10.6_(an)_; −12.1; −12.8; −11.2
Piroxicam	AR	−9.3 and −9.0_(an)_
Plerixafor	ERα; ERβ	−10.8_(an)_; −10.9_(an)_
Pomalidomide	AR	−8.7
Ponatinib	GR; PPARγ	−10.0_(an)_; −10.6
Protriptyline	ERβ	−9.1_(an)_
Regorafenib	ERα; ERβ	−10.0; −9.7_(an)_
Revefenacin	ERβ; GR; PPARγ	−9.7_(an)_; −10.2_(an)_; −10.7
Risperidone	ERα; ERβ; LXRβ	−9.7_(an)_; −9.5_(an)_; −12.3
Rolapitant	ERβ; PPARγ; TRβ	−10.5_(an)_; −10.5; −10.8
Sertraline	ERα	−9.5
Sitagliptin	ERα	−9.5
Solifenacin	ERβ	−9.6_(an)_
Sonidegib	ERβ	−9.5_(an)_
Sorafenib	ERα	−9.9_(an)_
Tadalafil	ERα; ERβ	−9.7_(an)_; −9.6_(an)_
Telmisartan	ERα; ERβ; PPARγ	−10.0_(an)_; −10.1_(an)_; −10.6
Telotristat	PPARγ	−10.4
Temazepam	ERα	−9.8
Thalidomide	AR	−8.7
Tolnaftate	ERα	−9.5
Tolvaptan	ERβ; GR	−9.6_(an)_; −10.3_(an)_
Triamterene	AR	−8.8 and −9.0_(an)_
Triazolam	ERβ	−9.1_(an)_
Trihexyphenidyl	AR; ERβ	−9.0_(an)_; −9.3
Umeclidinium	ERα	−9.5
Vilazodone	GR	−10.8
Vorapaxar	ERβ; GR	−9.7_(an)_; −10.6_(an)_
Voriconazole	AR	−8.8_(an)_
Vortioxetine	AR	−8.5_(an)_
Warfarin	ERα	−9.4_(an)_
Zafirlukast	ERβ; PPARγ	−9.7_(an)_; −10.9

^1^ The table shows medicines that do not bind to a nuclear receptor as their pharmacological mode of action. ^2^AR—androgen receptor; ERα—estrogen receptor; ERβ—estrogen receptor β; GR—glucocorticoid receptor; LXRα—liver X receptor α; LXRβ—liver X receptor β; PPARα—peroxisome proliferator-activated receptor α; PPARβ—peroxisome proliferator-activated receptor β; PPARγ—peroxisome proliferator-activated receptor γ; RXRα—retinoid X receptor α; TRα—thyroid receptor α; TRβ—thyroid receptor β; an—antagonist conformation.

## Supplementary Materials

The following are available online at https://www.mdpi.com/1420-3049/25/7/1616/s1: Table S1: 1046 US-approved and marketed drugs with Endocrine Disruptome (ED)-predicted binding to respective nuclear receptors and Table S2: Structure_information.pdf.
