# Supplementary material for: Computational Study of Drugs Targeting Nuclear Receptors
Source: Molecules. 2020 Apr 1;25(7):1616. doi: 10.3390/molecules25071616 (PMC7180905; doi:10.3390/molecules25071616)
Supplement: Supplementary file 1 [file molecules-25-01616-s001.pdf]

Computational study of drugs targeting nuclear receptors

Deleted: endocrine-disrupting potential of medicines

Supplementary material

Table S1. 1046 US-approved and marketed drugs with Endocrine Disruptome (ED)-predicted binding to respective nuclear receptors[1].

Deleted: medicines

No.	Name	AR	ARan.	ERα	ERαan.	ERβ	ERβan.	GR	GRan.	LXRα	LXRβ	PPARα	PPARβ	PPARγ	RXRα	TRα	TRβ
1	.ALPHA.-TOCOPHEROL, D-	1	2	2	1	1	1	2	1	1	1	2	1	1	2	1	1
2	2-MERCAPTOETHANESULFONIC ACID	1	2	1	1	1	1	1	1	1	1	1	1	1	1	1	1
3	21-DESACETYLDEFLAZACORT	1	1	1	1	1	1	2	2	1	3	1	1	1	1	1	1
4	4-HYDROXYBUTANOIC ACID	1	2	1	1	1	1	1	1	1	1	1	1	1	1	1	1
5	4-HYDROXYCYCLOPHOSPHAMIDE	1	2	1	1	1	1	1	1	1	1	1	1	1	1	1	1
6	4-HYDROXYIFOSFAMIDE	1	2	1	1	1	1	1	1	1	1	1	1	1	1	1	1
7	5'-DEOXY-5-FLUOROCYTIDINE	2	3	1	1	1	1	1	1	1	1	1	1	1	1	1	1
8	ABACAVIR	1	3	1	1	1	1	2	1	1	1	1	1	1	1	2	1
9	ABEMACICLIB	1	1	1	2	1	1	3	2	2	2	2	1	1	1	1	1
10	ABIRATERONE	1	2	1	1	1	1	3	3	2	3	1	1	1	1	1	4
11	ACALABRUTINIB	1	1	1	1	1	1	2	3	3	2	3	1	2	1	1	1
12	ACAMPROSATE	1	2	1	1	1	1	1	1	1	1	1	1	1	1	1	1
13	ACEBUTOLOL	1	2	1	1	1	1	2	1	1	1	1	1	1	1	2	2
14	ACETAMINOPHEN	1	2	1	1	1	1	1	1	1	1	1	1	1	1	1	1
15	ACETAZOLAMIDE	1	2	1	1	1	1	1	1	1	1	1	1	1	1	1	1
16	ACETOHYDROXAMIC ACID	1	2	1	1	1	1	1	1	1	1	1	1	1	1	1	1
17	ACETYLCHOLINE	1	2	1	1	1	1	1	1	1	1	1	1	1	1	1	1
18	ACETYLCYSTEINE	1	2	1	1	1	1	1	1	1	1	1	1	1	1	1	1
19	ACITRETIN	1	1	1	1	1	1	2	1	1	1	1	1	1	1	2	3
20	ACLIDINIUM	2	1	1	1	1	1	2	1	2	1	1	1	1	1	1	1
21	ACRIVASTINE	1	2	1	2	1	3	2	1	1	1	1	1	2	1	1	2
22	ACYCLOVIR	1	2	1	1	1	1	1	1	1	1	1	1	1	1	1	1

No.	Name	AR	ARan.	ERα	ERαan.	ERβ	ERβan.	GR	GRan.	LXRα	LXRβ	PPARα	PPARβ	PPARγ	RXRα	TRα	TRβ
23	ADAPALENE	1	1	1	3	1	3	3	3	4	4	1	1	3	1	1	1
24	ADEFOVIR	1	2	1	1	1	1	1	1	1	1	1	1	1	1	1	1
25	AFATINIB	1	1	1	1	1	1	2	2	2	2	3	1	3	1	2	1
26	ALANINE	1	2	1	1	1	1	1	1	1	1	1	1	1	1	1	1
27	ALBENDAZOLE	1	2	1	1	1	1	1	1	1	1	1	1	1	1	1	1
28	ALBUTEROL	1	2	1	1	1	1	1	1	1	1	1	1	1	1	2	1
29	ALCAFTADINE	1	2	1	1	1	3	2	1	1	1	1	1	1	1	1	1
30	ALCLOMETASONE	1	2	1	1	1	1	1	1	1	1	1	1	1	1	1	1
31	ALECTINIB	1	1	1	1	1	1	1	2	1	1	1	1	1	1	1	1
32	ALENDRONIC ACID	1	2	1	1	1	1	1	1	1	1	1	1	1	1	1	1
33	ALFENTANIL	1	2	1	1	1	1	2	1	1	1	1	1	1	1	1	2
34	ALFUZOSIN	1	2	1	1	1	1	2	1	1	1	1	1	1	1	2	2
35	ALISKIREN	1	1	1	1	1	1	2	1	1	1	1	1	1	1	1	1
36	ALITRETINOIN	1	2	1	2	1	1	2	1	1	1	1	1	1	2	2	1
37	ALLANTOIN	1	2	1	1	1	1	1	1	1	1	1	1	1	1	1	1
38	ALLOPURINOL	1	2	1	1	1	1	1	1	1	1	1	1	1	1	1	1
39	ALMOTRIPTAN	1	2	1	1	1	1	2	1	1	1	1	1	1	1	2	2
40	ALOGLIPTIN	1	2	2	1	1	1	2	1	1	1	1	1	1	1	1	1
41	ALOSETRON	1	3	1	1	1	1	2	1	1	1	1	1	1	1	2	2
42	ALPRAZOLAM	1	2	2	2	2	2	2	2	1	1	1	1	1	1	2	1
43	ALPROSTADIL	1	2	1	1	1	1	2	1	1	1	1	1	1	1	1	1
44	ALVIMOPAN	1	2	1	1	1	1	2	2	1	2	1	1	1	1	1	2
45	AMANTADINE	1	2	1	1	1	1	1	1	1	1	1	1	1	1	1	1
46	AMBRISENTAN	1	2	1	1	1	1	2	1	1	1	1	1	1	1	1	2
47	AMCINONIDE	1	1	1	1	1	1	2	3	1	1	1	1	1	1	1	1
48	AMIFAMPRIDINE	1	2	1	1	1	1	1	1	1	1	1	1	1	1	1	1
49	AMIFOSTINE	1	2	1	1	1	1	1	1	1	1	1	1	1	1	1	1
50	AMIKACIN	1	1	1	1	1	1	1	1	1	1	1	1	1	1	1	1
51	AMILORIDE	1	2	1	1	1	1	1	1	1	1	1	1	1	1	1	1

No.	Name	AR	ARan.	ERα	ERαan.	ERβ	ERβan.	GR	GRan.	LXRα	LXRβ	PPARα	PPARβ	PPARγ	RXRα	TRα	TRβ
52	AMINOCAPROIC ACID	1	2	1	1	1	1	1	1	1	1	1	1	1	1	1	1
53	AMINOLEVULINIC ACID	1	2	1	1	1	1	1	1	1	1	1	1	1	1	1	1
54	AMINOSALICYLIC ACID	1	2	1	1	1	1	1	1	1	1	1	1	1	1	1	1
55	AMITRIPTYLINE	1	2	3	2	2	4	2	1	2	1	1	1	1	1	2	3
56	AMLODIPINE	1	2	1	1	1	1	1	1	1	1	1	1	1	1	1	1
57	AMOXAPINE	1	3	3	1	2	3	2	2	1	1	1	1	1	1	1	1
58	AMOXICILLIN	1	4	1	1	1	1	2	1	1	1	1	1	1	1	1	1
59	AMPHETAMINE	1	2	1	1	1	1	1	1	1	1	1	1	1	1	1	1
60	AMPICILLIN	1	2	1	1	1	1	2	1	1	1	1	1	1	1	1	1
61	AMPRENAVIR	1	2	3	1	1	1	2	1	1	1	1	1	1	1	1	2
62	ANAGRELIDE	2	3	1	1	1	1	2	1	1	1	1	1	1	1	1	1
63	ANASTROZOLE	1	2	1	1	1	1	1	1	1	1	1	1	1	1	1	1
64	ANHYDROUS CITRIC ACID	1	2	1	1	1	1	1	1	1	1	1	1	1	1	1	1
65	ANHYDROUS DEXTROSE	1	2	1	1	1	1	1	1	1	1	1	1	1	1	1	1
66	APALUTAMIDE	1	1	1	1	1	1	2	1	2	3	1	1	1	1	1	1
67	APIXABAN	1	1	1	1	1	3	1	3	1	1	1	1	4	1	1	1
68	APRACLONIDINE	1	2	1	1	1	1	1	1	1	1	1	1	1	1	1	1
69	APREMILAST	1	2	1	1	1	1	2	2	1	1	1	1	1	1	1	1
70	APREPITANT	1	1	1	2	1	3	2	4	3	4	3	4	4	1	1	2
71	AR-13503	1	2	1	2	1	3	2	1	1	1	1	1	1	1	1	1
72	ARFORMOTEROL	1	2	1	1	1	1	2	1	1	1	1	1	1	1	2	2
73	ARGININE	1	2	1	1	1	1	1	1	1	1	1	1	1	1	1	1
74	ARIPIPRAZOLE	1	2	3	2	1	1	2	2	2	2	3	2	2	2	1	1
75	ARMODAFINIL	1	3	2	1	2	1	2	1	1	1	1	1	1	1	2	2
76	ARTEMETHER	2	4	3	1	4	3	2	1	2	1	1	1	1	1	1	1
77	ARTICAINE	1	2	1	1	1	1	1	1	1	1	1	1	1	1	1	1
78	ASENAPINE	1	2	4	1	1	3	2	2	1	1	1	1	1	1	1	1
79	ASPIRIN	1	2	1	1	1	1	1	1	1	1	1	1	1	1	1	1
80	ATENOLOL	1	2	1	1	1	1	2	1	1	1	1	1	1	1	2	1

No.	Name	AR	ARan.	ERα	ERαan.	ERβ	ERβan.	GR	GRan.	LXRα	LXRβ	PPARα	PPARβ	PPARγ	RXRα	TRα	TRβ
81	ATOMOXETINE	1	3	1	1	1	1	2	1	1	1	1	1	1	1	2	2
82	ATORVASTATIN	1	1	1	1	1	1	2	1	1	1	1	1	1	1	1	1
83	ATOVAQUONE	1	1	1	1	1	1	4	2	4	4	1	1	1	1	2	4
84	ATROPINE	2	3	1	1	1	1	2	1	1	1	1	1	1	1	2	2
85	AVANAFIL	1	1	1	1	1	1	1	2	1	1	1	1	1	1	1	1
86	AVIBACTAM	2	2	1	1	1	1	1	1	1	1	1	1	1	1	1	1
87	AVOBENZONE	1	2	1	1	2	1	2	1	1	1	1	1	1	1	3	3
88	AXITINIB	1	1	2	2	1	3	4	2	2	2	1	1	1	1	2	2
89	AZACITIDINE	1	2	1	1	1	1	1	1	1	1	1	1	1	1	1	1
90	AZATHIOPRINE	1	2	1	1	1	1	1	1	1	1	1	1	1	1	2	1
91	AZELAIC ACID	1	2	1	1	1	1	1	1	1	1	1	1	1	1	1	1
92	AZELASTINE	1	2	1	2	1	1	2	2	3	2	3	1	3	2	1	1
93	AZILSARTAN	1	1	1	2	1	4	2	4	2	2	2	2	2	1	1	1
94	AZTREONAM	1	2	1	1	1	1	2	1	1	1	1	1	1	1	1	1
95	BACLOFEN	1	2	1	1	1	1	1	1	1	1	1	1	1	1	1	1
96	BALSALAZIDE	1	1	1	1	1	1	2	1	1	1	1	1	1	1	2	1
97	BARICITINIB	1	2	1	1	1	1	2	1	1	1	1	1	1	1	2	2
98	BAZEDOXIFENE	1	1	4	4	1	4	2	3	1	2	1	1	3	1	1	1
99	BECLOMETHASONE	1	2	1	1	1	1	2	1	1	1	1	1	1	1	1	1
100	BEDAQUILINE	1	1	1	1	1	2	1	1	1	1	1	1	1	1	1	1
101	BELINOSTAT	1	3	2	1	3	1	2	2	1	1	1	1	1	1	4	3
102	BENAZEPRILAT	1	2	1	1	1	1	2	1	1	1	1	1	1	2	2	2
103	BENDAMUSTINE	1	2	1	1	1	1	2	1	1	1	1	1	1	1	1	1
104	BENDROFLUMETHIAZIDE	1	2	2	1	2	3	3	2	2	2	2	1	1	1	1	1
105	BENOXINATE	1	2	1	1	1	1	1	1	1	1	1	1	1	1	1	1
106	BENZNIDAZOLE	2	2	1	1	2	1	2	1	1	1	1	1	1	1	2	2
107	BENZOCAINE	1	2	1	1	1	1	1	1	1	1	1	1	1	1	1	1
108	BENZOIC ACID	1	2	1	1	1	1	1	1	1	1	1	1	1	1	1	1
109	BENZOYL PEROXIDE	1	2	1	1	1	1	1	1	1	1	1	1	1	1	2	2

No.	Name	AR	ARan.	ERα	ERαan.	ERβ	ERβan.	GR	GRan.	LXRα	LXRβ	PPARα	PPARβ	PPARγ	RXRα	TRα	TRβ
110	BENZPHETAMINE	1	2	1	1	1	1	1	1	1	1	1	1	1	1	2	2
111	BENZATROPINE	1	2	4	2	3	4	2	2	2	1	1	1	2	1	2	2
112	BENZYL ALCOHOL	1	2	1	1	1	1	1	1	1	1	1	1	1	1	1	1
113	BEPOTASTINE	1	2	1	1	1	1	2	1	1	1	1	1	2	1	2	2
114	BETAINE	1	2	1	1	1	1	1	1	1	1	1	1	1	1	1	1
115	BETAMETHASONE	2	2	1	1	1	1	2	1	1	1	1	1	1	1	1	2
116	BETAXOLOL	1	2	1	1	1	1	1	1	1	1	1	1	1	1	2	1
117	BETHANECHOL	1	2	1	1	1	1	1	1	1	1	1	1	1	1	1	1
118	BETRIXABAN	1	1	1	2	1	1	2	2	2	2	2	1	1	1	1	1
119	BEXAROTENE	1	2	1	1	1	2	2	2	2	3	1	2	2	4	1	2
120	BICALUTAMIDE	1	3	3	1	2	1	2	1	2	1	1	1	1	1	2	3
121	BICARBONATE ION	1	1	1	1	1	1	1	1	1	1	1	1	1	1	1	1
122	BICTEGRAVIR	1	2	1	1	1	1	3	3	3	3	1	3	2	2	3	2
123	BIMATOPROST	1	2	2	1	1	1	2	1	1	1	1	1	1	1	2	2
124	BINIMETINIB	1	2	1	1	1	1	2	1	1	1	1	1	1	1	1	1
125	BIOTIN	1	2	1	1	1	1	1	1	1	1	1	1	1	1	1	1
126	BISOPROLOL	1	2	1	1	1	1	2	1	1	1	1	1	1	1	2	1
127	BOSENTAN	1	1	1	1	1	1	1	1	1	1	1	1	1	1	1	1
128	BOSUTINIB	1	1	1	1	1	2	1	2	1	1	1	1	1	1	1	1
129	BRETYLIUM	1	2	1	1	1	1	1	1	1	1	1	1	1	1	1	1
130	BREXPIPRAZOLE	1	2	4	2	1	3	3	3	2	3	3	2	2	3	1	2
131	BRIGATINIB	1	1	1	2	1	1	2	3	1	1	1	1	1	1	1	1
132	BRIMONIDINE	2	3	1	1	1	1	2	1	1	1	1	1	1	1	2	1
133	BRINZOLAMIDE	1	2	1	1	1	1	1	1	1	1	1	1	1	1	1	1
134	BRIVARACETAM	1	2	1	1	1	1	1	1	1	1	1	1	1	1	1	1
135	BROMFENAC	1	2	2	1	1	1	2	1	1	1	1	1	1	1	2	2
136	BROMPHENIRAMINE	1	2	1	1	1	1	1	1	1	1	1	1	1	1	1	1
137	BUMETANIDE	1	2	1	1	1	1	2	1	1	1	1	1	1	1	1	1
138	BUPIVACAINE	1	3	2	1	1	1	2	1	1	1	1	1	1	1	2	2

No.	Name	AR	ARan.	ERα	ERαan.	ERβ	ERβan.	GR	GRan.	LXRα	LXRβ	PPARα	PPARβ	PPARγ	RXRα	TRα	TRβ
139	BUPRENORPHINE	1	2	1	1	1	1	1	1	1	1	1	1	1	1	1	1
140	BUPROPION	1	2	1	1	1	1	1	1	1	1	1	1	1	1	2	1
141	BUSPIRONE	1	2	1	1	1	1	2	1	1	1	2	1	1	1	1	2
142	BUSULFAN	1	2	1	1	1	1	1	1	1	1	1	1	1	1	1	1
143	BUTALBITAL	1	2	1	1	1	1	1	1	1	1	1	1	1	1	1	1
144	BUTENAFINE	1	3	2	3	1	4	2	2	3	2	1	3	2	1	2	3
145	BUTOCONAZOLE	1	2	1	1	1	1	2	1	1	1	1	1	1	1	1	1
146	BUTORPHANOL	3	3	4	2	2	1	2	1	1	1	1	1	1	1	1	2
147	CABERGOLINE	1	1	1	1	1	1	2	1	1	1	1	1	1	1	1	1
148	CABOZANTINIB	1	1	1	3	1	4	2	4	3	2	1	1	2	1	1	1
149	CAFFEINE	1	2	1	1	1	1	1	1	1	1	1	1	1	1	1	1
150	CALCIFEDIOL	1	1	1	1	1	1	2	1	3	2	1	1	1	1	2	3
151	CALCITRIOL	1	2	1	1	1	1	2	2	2	2	1	1	1	1	2	2
152	CAMPHOR (SYNTHETIC)	1	2	1	1	1	1	1	1	1	1	1	1	1	1	1	1
153	CANAGLIFLOZIN	1	2	1	1	1	1	2	3	2	2	2	4	1	2	1	2
154	CANDESARTAN	1	1	1	3	1	4	2	3	1	2	1	3	2	1	1	1
155	CANNABIDIOL	1	3	1	1	1	1	2	1	1	1	1	1	1	1	2	2
156	CAPSAICIN	1	2	1	1	1	1	2	1	1	1	1	1	1	1	2	2
157	CAPTOPRIL	1	2	1	1	1	1	1	1	1	1	1	1	1	1	1	1
158	CARBAMAZEPINE	3	4	4	2	3	4	2	2	1	1	1	1	1	1	2	2
159	CARBAMOYLCHOLINE	1	2	1	1	1	1	1	1	1	1	1	1	1	1	1	1
160	CARBIDOPA	1	2	1	1	1	1	1	1	1	1	1	1	1	1	1	1
161	CARBONATE ION	1	1	1	1	1	1	1	1	1	1	1	1	1	1	1	1
162	CARBOPROST	1	2	1	1	1	1	1	1	1	1	1	1	1	1	1	1
163	CARGLUMIC ACID	1	2	1	1	1	1	1	1	1	1	1	1	1	1	1	1
164	CARIPRAZINE	1	2	1	2	1	1	2	3	2	2	1	1	1	1	1	2
165	CARISOPRODOL	1	2	1	1	1	1	1	1	1	1	1	1	1	1	1	1
166	CARMUSTINE	1	2	1	1	1	1	1	1	1	1	1	1	1	1	1	1
167	CARTEOLOL	2	3	1	1	1	1	2	1	1	1	1	1	1	1	2	2

No.	Name	AR	ARan.	ERα	ERαan.	ERβ	ERβan.	GR	GRan.	LXRα	LXRβ	PPARα	PPARβ	PPARγ	RXRα	TRα	TRβ
168	CARVEDILOL	1	2	1	1	1	1	2	1	2	1	2	1	1	1	2	1
169	CEFACLOR	1	2	1	1	1	1	2	1	1	1	1	1	1	1	1	2
170	CEFADROXIL	1	2	1	1	1	2	1	1	1	1	1	1	1	1	2	2
171	CEFAZOLIN	1	2	1	1	1	1	2	1	1	1	1	1	1	1	1	1
172	CEFDINIR	1	2	1	1	1	1	2	1	1	1	1	1	1	1	1	1
173	CEFEPIME	1	1	1	1	1	1	2	1	1	1	1	1	1	1	1	1
174	CEFIXIME	1	2	1	1	1	1	1	1	1	1	1	1	1	1	1	2
175	CEFOTAXIME	1	2	1	1	1	1	1	1	1	1	1	1	1	1	2	1
176	CEFOTETAN	1	1	1	1	1	1	2	1	1	1	1	1	1	1	1	1
177	CEFOXITIN	1	2	1	1	1	1	2	1	1	1	1	1	1	1	1	2
178	CEFPODOXIME	1	2	1	1	1	1	2	1	1	1	1	1	1	1	2	1
179	CEFTAZIDIME	1	1	1	1	1	1	2	1	1	1	1	1	1	1	1	1
180	CEFTRIAXONE	1	1	1	1	1	1	2	1	1	1	1	1	1	1	1	1
181	CEFUROXIME	1	2	1	1	1	1	2	1	1	1	1	1	1	1	1	2
182	CELECOXIB	1	2	4	3	1	4	1	2	2	1	1	1	2	2	1	1
183	CEPHALEXIN	1	2	1	1	1	1	2	1	1	1	1	1	1	1	1	2
184	CERITINIB	1	1	1	2	1	4	2	3	2	2	3	1	2	1	1	1
185	CETIRIZINE	1	2	1	1	1	1	2	1	1	1	1	1	1	1	1	2
186	CEVIMELINE	1	2	1	1	1	1	1	1	1	1	1	1	1	1	1	1
187	CHLOPHEDIANOL	1	2	1	1	1	1	2	1	1	1	1	1	1	1	2	1
188	CHLORAMBUCIL	1	2	1	1	1	1	1	1	1	1	1	1	1	1	1	1
189	CHLORAMPHENICOL	1	2	1	1	1	1	1	1	1	1	1	1	1	1	2	1
190	CHLORCYCLIZINE	1	3	3	2	1	3	1	1	1	1	1	1	1	1	1	2
191	CHLORDIAZEPOXIDE	1	2	3	1	1	1	2	1	1	1	1	1	1	1	1	1
192	CHLORHEXIDINE	1	2	1	1	1	1	2	1	1	1	2	1	1	1	2	1
193	CHLOROPROCAINE	1	2	1	1	1	1	1	1	1	1	1	1	1	1	1	1
194	CHLOROQUINE	1	2	1	1	1	1	2	1	1	1	1	1	1	1	2	2
195	CHLOROTHIAZIDE	1	2	1	1	1	1	1	1	1	1	1	1	1	1	1	1
196	CHLORPHENIRAMINE	1	2	1	1	1	1	1	1	1	1	1	1	1	1	2	2

No.	Name	AR	ARan.	ERα	ERαan.	ERβ	ERβan.	GR	GRan.	LXRα	LXRβ	PPARα	PPARβ	PPARγ	RXRα	TRα	TRβ
197	CHLORPROMAZINE	1	2	1	1	1	1	2	1	1	1	1	1	1	1	1	2
198	CHLORTETRACYCLINE	1	1	1	1	1	1	1	1	1	1	1	1	1	1	1	1
199	CHLORTHALIDONE	2	3	1	2	1	1	2	1	1	1	1	1	1	1	3	2
200	CHLORZOXAZONE	1	2	1	1	1	1	1	1	1	1	1	1	1	1	1	1
201	CHOLECALCIFEROL	1	2	1	2	1	1	2	1	2	3	1	1	1	1	2	3
202	CHOLIC ACID	1	2	1	1	1	1	2	1	1	1	1	1	1	1	1	1
203	CHOLINE C-11	1	2	1	1	1	1	1	1	1	1	1	1	1	1	1	1
204	CICLESONIDE	1	1	1	1	1	1	1	1	1	1	1	1	1	1	1	1
205	CICLOPIROX	2	3	1	1	1	1	1	1	1	1	1	1	1	1	2	1
206	CIDOFOVIR	1	2	1	1	1	1	1	1	1	1	1	1	1	1	1	1
207	CILASTATIN	1	2	1	1	1	1	1	1	1	1	1	1	1	1	2	1
208	CILOSTAZOL	2	3	2	1	1	1	3	2	2	2	1	1	2	1	2	3
209	CIMETIDINE	1	2	1	1	1	1	1	1	1	1	1	1	1	1	1	1
210	CINACALCET	1	4	4	2	3	4	2	1	3	2	1	3	2	2	4	3
211	CIPROFLOXACIN	2	2	1	1	1	1	2	1	1	1	1	1	1	1	2	3
212	CITALOPRAM	1	2	2	1	1	1	2	1	1	1	1	1	1	1	1	2
213	CLADRIBINE	2	3	1	1	1	1	1	1	1	1	1	1	1	1	2	1
214	CLAVULANIC ACID	1	2	1	1	1	1	1	1	1	1	1	1	1	1	1	1
215	CLEMASTINE	1	3	3	3	2	2	2	1	2	1	1	1	1	1	2	2
216	CLEVIDIPINE	1	2	2	1	1	1	1	1	1	1	1	1	1	1	1	1
217	CLIDINIUM	1	2	3	2	2	3	2	1	2	1	1	1	1	1	2	2
218	CLINDAMYCIN	1	1	1	1	1	1	2	1	1	1	1	1	1	1	1	1
219	CLIOQUINOL	1	2	1	1	1	1	1	1	1	1	1	1	1	1	1	1
220	CLOBAZAM	1	2	2	1	1	1	2	1	1	1	1	1	1	1	1	1
221	CLOBETASOL	1	2	1	1	1	1	2	1	1	2	1	1	1	1	1	1
222	CLOCORTOLONE	2	2	1	1	1	1	2	1	1	1	1	1	1	1	1	1
223	CLOFARABINE	2	3	1	1	1	1	1	1	1	1	1	1	1	1	2	1
224	CLOMIPRAMINE	1	3	2	2	1	1	2	1	1	1	1	1	1	1	2	2
225	CLONAZEPAM	1	1	2	2	3	4	2	2	1	1	1	1	1	1	1	2

No.	Name	AR	ARan.	ERα	ERαan.	ERβ	ERβan.	GR	GRan.	LXRα	LXRβ	PPARα	PPARβ	PPARγ	RXRα	TRα	TRβ
226	CLONIDINE	1	2	1	1	1	1	1	1	1	1	1	1	1	1	1	1
227	CLOPIDOGREL	1	3	1	1	1	1	2	1	1	1	1	1	1	1	1	2
228	CLORAZEPATE	1	2	4	2	2	3	2	1	1	1	1	1	1	1	1	1
229	CLOTRIMAZOLE	1	1	1	1	1	1	2	1	1	1	1	1	1	1	1	1
230	CLOZAPINE	1	2	1	1	1	1	1	1	1	1	1	1	1	1	1	1
231	COBIMETINIB	1	2	1	1	1	1	3	2	3	2	1	1	2	1	1	1
232	COCAINE	2	4	2	1	2	1	2	1	1	1	1	1	1	1	2	2
233	CODEINE	4	4	2	2	2	3	2	1	1	1	1	1	1	1	1	2
234	COLCHICINE	1	1	1	1	1	1	1	1	1	1	1	1	1	1	1	1
235	CONIVAPTAN	1	1	1	1	1	4	4	4	1	1	1	2	2	1	1	1
236	COPANLISIB	1	1	1	1	1	1	1	1	1	1	1	1	1	1	1	1
237	COPPER	1	1	1	1	1	1	1	1	1	1	1	1	1	1	1	1
238	CORTISONE	2	3	1	1	1	1	2	2	1	2	1	1	1	1	1	2
239	CRIZOTINIB	1	2	1	2	1	1	2	3	2	2	1	1	1	1	2	2
240	CROMOLYN	1	2	2	1	1	1	3	2	1	2	3	1	3	1	1	1
241	CUPRIC CATION	1	1	1	1	1	1	1	1	1	1	1	1	1	1	1	1
242	CYCLIZINE	1	3	3	1	3	3	2	1	1	1	1	1	1	1	1	1
243	CYCLOBENZAPRINE	1	4	4	2	3	4	2	2	1	1	2	1	1	1	1	2
244	CYCLOPENTOLATE	1	2	1	1	1	1	1	1	1	1	1	1	1	1	1	2
245	CYCLOSERINE	1	2	1	1	1	1	1	1	1	1	1	1	1	1	1	1
246	CYPROHEPTADINE	1	2	4	3	2	4	2	3	2	2	1	1	1	1	1	2
247	CYSTEAMINE	1	1	1	1	1	1	1	1	1	1	1	1	1	1	1	1
248	CYSTEINE	1	2	1	1	1	1	1	1	1	1	1	1	1	1	1	1
249	CYTARABINE	1	2	1	1	1	1	1	1	1	1	1	1	1	1	1	1
250	DABRAFENIB	1	1	1	3	1	4	2	3	3	2	1	1	3	1	1	1
251	DACARBAZINE	1	2	1	1	1	1	1	1	1	1	1	1	1	1	1	1
252	DACOMITINIB	1	1	1	1	1	1	2	4	1	1	1	1	1	1	1	1
253	DALFAMPRIDINE	1	2	1	1	1	1	1	1	1	1	1	1	1	1	1	1
254	DANAZOL	1	2	1	1	1	1	2	2	3	1	1	1	1	1	1	2

No.	Name	AR	ARan.	ERα	ERαan.	ERβ	ERβan.	GR	GRan.	LXRα	LXRβ	PPARα	PPARβ	PPARγ	RXRα	TRα	TRβ
255	DANTROLENE	1	1	1	1	1	1	2	1	1	1	1	1	1	1	2	2
256	DAPAGLIFLOZIN	1	1	1	1	1	1	2	1	1	1	1	1	1	1	2	2
257	DAPSONE	1	2	1	1	1	1	1	1	1	1	1	1	1	1	2	2
258	DARIFENACIN	1	2	4	3	1	3	3	3	2	2	4	2	2	2	1	2
259	DARUNAVIR	1	1	1	1	1	2	2	1	1	1	1	1	2	1	1	1
260	DASABUVIR	1	1	1	1	1	1	2	3	1	1	1	1	1	1	1	1
261	DASATINIB	1	1	1	3	1	2	2	2	1	1	1	1	1	1	1	1
262	DAUNORUBICIN	1	1	1	1	1	1	1	1	1	1	1	1	2	1	1	1
263	DEACETYLBISACODYL	1	2	4	3	2	4	2	1	1	1	1	1	1	1	2	2
264	DECITABINE	1	2	1	1	1	1	1	1	1	1	1	1	1	1	1	1
265	DEFERASIROX	1	1	1	4	1	3	2	2	3	2	1	1	2	2	1	2
266	DEFERIPRONE	1	2	1	1	1	1	1	1	1	1	1	1	1	1	1	1
267	DEFEROXAMINE	1	2	1	1	1	1	2	1	1	1	1	1	1	1	1	1
268	DELAFLOXACIN	1	1	1	1	1	1	2	1	1	1	1	1	1	1	1	1
269	DEMECLOCYCLINE	1	1	1	1	1	1	1	1	1	1	1	1	1	1	1	1
270	DEOXYCHOLIC ACID	1	2	1	1	2	1	2	1	2	2	1	1	1	1	1	1
271	DESFLURANE	1	2	1	1	1	1	1	1	1	1	1	1	1	1	1	1
272	DESIPRAMINE	2	3	3	1	1	2	2	1	1	1	1	1	1	1	2	2
273	DESLORATADINE	1	1	3	1	1	2	2	2	1	1	2	1	1	1	1	1
274	DESOGESTREL	4	4	3	1	4	1	2	1	3	2	1	1	1	1	2	2
275	DESONIDE	1	1	1	1	1	1	2	1	1	2	1	1	1	1	1	1
276	DESOXIMETASONE	1	2	1	1	1	1	2	1	1	2	1	1	1	1	2	2
277	DESVENLAFAXINE	2	2	1	1	1	1	2	1	1	1	1	1	1	1	2	1
278	DEUTETRABENAZINE	1	2	1	1	1	1	2	1	1	1	1	1	1	1	1	2
279	DEXAMETHASONE	1	2	1	1	1	1	2	2	1	2	1	1	1	1	1	1
280	DEXBROMPHENIRAMINE	1	2	1	1	1	1	1	1	1	1	1	1	1	1	1	1
281	DEXCHLORPHENIRAMINE	1	2	1	1	1	1	1	1	1	1	1	1	1	1	1	1
282	DEXMEDETOMIDINE	1	2	1	1	1	1	1	1	1	1	1	1	1	1	2	1
283	DEXMETHYLPHENIDATE	1	2	1	1	1	1	1	1	1	1	1	1	1	1	1	1

No.	Name	AR	ARan.	ERα	ERαan.	ERβ	ERβan.	GR	GRan.	LXRα	LXRβ	PPARα	PPARβ	PPARγ	RXRα	TRα	TRβ
284	DEXRAZOXANE	2	2	1	1	1	1	2	1	1	1	1	1	1	1	2	2
285	DEXTROAMPHETAMINE	1	2	1	1	1	1	1	1	1	1	1	1	1	1	1	1
286	DEXTROMETHORPHAN	1	2	3	1	1	1	2	1	1	1	1	1	1	1	2	3
287	DIAZEPAM	1	3	4	1	2	2	2	1	1	1	1	1	1	1	1	1
288	DIAZOXIDE	1	2	1	1	1	1	1	1	1	1	1	1	1	1	2	1
289	DIBUCAINE	1	2	1	1	1	1	2	1	1	1	1	1	1	1	2	2
290	DICLOFENAC	2	3	1	1	2	1	2	1	1	1	1	1	1	1	2	2
291	DICLOXACILLIN	1	1	1	1	1	1	2	1	1	2	1	1	1	1	1	1
292	DICYCLOMINE	2	3	1	1	1	1	1	1	1	1	1	1	1	1	1	2
293	DIDANOSINE	2	1	1	1	1	1	1	1	1	1	1	1	1	1	2	1
294	DIENOGEST	3	3	2	1	1	1	2	1	2	1	1	1	1	1	2	2
295	DIETHYLPROPION	1	2	1	1	1	1	1	1	1	1	1	1	1	1	1	1
296	DIFLORASONE	1	2	1	1	1	1	3	1	2	2	1	1	1	1	1	1
297	DIFLUNISAL	3	3	2	1	2	2	2	1	1	1	1	1	1	1	3	2
298	DIFLUPREDNATE	1	1	1	1	1	1	1	1	1	1	1	1	1	1	1	1
299	DIHYDROCODEINE	1	3	3	1	2	3	2	1	1	1	1	1	1	1	1	2
300	DIHYDROERGOTAMINE	1	1	1	4	1	4	1	4	1	1	1	1	1	1	1	1
301	DILTIAZEM	1	1	1	1	1	1	1	1	1	1	1	1	1	1	1	1
302	DIMERCAPROL	1	2	1	1	1	1	1	1	1	1	1	1	1	1	1	1
303	DIMETHYL SULFOXIDE	1	1	1	1	1	1	1	1	1	1	1	1	1	1	1	1
304	DINOPROSTONE	1	2	1	1	1	1	2	1	1	1	1	1	1	1	1	1
305	DIPHENHYDRAMINE	1	2	1	1	1	1	2	1	1	1	1	1	1	1	2	2
306	DIPHENOXYLATE	1	2	1	2	1	1	2	3	1	1	1	1	1	1	1	2
307	DIPYRIDAMOLE	1	1	1	1	1	1	1	1	1	1	1	1	1	1	1	1
308	DISOPYRAMIDE	1	2	1	1	1	1	2	1	1	1	1	1	1	1	1	2
309	DISULFIRAM	1	2	1	1	1	1	1	1	1	1	1	1	1	1	1	1
310	DOBUTAMINE	1	2	2	1	2	1	2	1	1	1	1	1	1	1	2	2
311	DOCOSANOL	1	2	1	1	1	1	1	1	1	1	1	1	1	1	1	1
312	DOFETILIDE	1	2	1	1	1	1	2	1	1	1	1	1	1	1	1	1

No.	Name	AR	ARan.	ERα	ERαan.	ERβ	ERβan.	GR	GRan.	LXRα	LXRβ	PPARα	PPARβ	PPARγ	RXRα	TRα	TRβ
313	DOLUTEGRAVIR	1	2	1	1	1	2	3	3	3	2	1	2	2	1	2	2
314	DONEPEZIL	1	1	2	2	1	1	2	2	2	2	1	1	1	1	2	3
315	DOPAMINE	1	1	1	1	1	1	1	1	1	1	1	1	1	1	1	1
316	DORAVIRINE	1	2	1	1	1	1	2	1	2	1	1	1	1	1	3	2
317	DORZOLAMIDE	1	1	1	1	1	1	1	1	1	1	1	1	1	1	1	1
318	DOXAPRAM	1	2	1	1	1	1	3	1	1	1	1	1	1	1	1	1
319	DOXAZOSIN	1	1	1	2	1	1	2	3	3	2	1	1	1	1	1	1
320	DOXERCALCIFEROL	1	1	1	1	1	1	3	2	3	2	1	1	1	1	1	1
321	DOXORUBICIN	1	1	1	1	1	1	1	2	1	2	1	1	2	1	1	1
322	DOXYLAMINE	1	2	1	1	1	1	2	1	1	1	1	1	1	1	2	2
323	DRONABINOL	1	2	1	1	2	1	2	1	1	1	1	1	1	1	2	2
324	DROPERIDOL	1	3	3	2	2	3	3	2	2	2	3	3	3	2	3	3
325	DROSPIRENONE	1	2	1	1	1	1	2	1	1	2	1	1	1	1	2	1
326	DROXIDOPA	1	2	1	1	1	1	1	1	1	1	1	1	1	1	1	1
327	DULOXETINE	2	3	2	1	1	1	2	1	1	1	1	1	1	1	2	2
328	DUTASTERIDE	1	1	1	2	1	1	1	2	1	1	1	1	1	1	1	1
329	DUVELISIB	1	2	2	2	1	4	2	3	4	4	2	1	1	1	1	1
330	DYCLONINE	1	2	1	1	1	1	2	1	1	1	1	1	1	1	2	2
331	ECAMSULE	1	1	2	3	1	1	1	1	3	1	1	1	1	1	1	1
332	ECHOTHIOPHATE	1	2	1	1	1	1	1	1	1	1	1	1	1	1	1	1
333	ECONAZOLE	1	2	1	1	1	2	2	1	1	1	1	1	2	1	2	2
334	EDARAVONE	1	3	1	1	1	1	1	1	1	1	1	1	1	1	2	1
335	EDETIC ACID	1	2	1	1	1	1	1	1	1	1	1	1	1	1	1	1
336	EDOXABAN	1	1	1	1	1	1	2	3	1	1	1	1	1	1	1	1
337	EFAVIRENZ	1	1	1	2	2	1	2	2	1	1	1	1	1	1	1	1
338	EFINACONAZOLE	2	4	3	1	3	2	2	1	1	1	1	1	1	1	1	2
339	EFLORNITHINE	1	2	1	1	1	1	1	1	1	1	1	1	1	1	1	1
340	ELETRIPTAN	1	3	1	1	1	1	2	1	2	2	1	2	2	1	1	2
341	ELIGLUSTAT	1	2	1	1	1	1	2	1	1	1	1	1	1	1	2	1

No.	Name	AR	ARan.	ERα	ERαan.	ERβ	ERβan.	GR	GRan.	LXRα	LXRβ	PPARα	PPARβ	PPARγ	RXRα	TRα	TRβ
342	ELTROMBOPAG	1	1	1	4	1	3	3	3	4	4	4	3	3	3	1	1
343	ELUXADOLINE	1	1	1	1	1	1	2	3	1	2	1	1	1	1	1	1
344	ELVITEGRAVIR	1	2	1	1	1	1	2	1	1	1	1	1	1	1	1	1
345	EMPAGLIFLOZIN	1	1	1	1	1	1	2	1	2	2	1	1	1	1	2	2
346	EMTRICITABINE	1	1	1	1	1	1	1	1	1	1	1	1	1	1	1	1
347	ENALAPRILAT	1	2	2	1	1	1	2	1	1	1	1	1	1	1	2	2
348	ENASIDENIB	1	1	1	1	1	1	2	2	2	3	4	1	2	1	1	1
349	ENTACAPONE	1	2	1	1	1	1	2	1	1	1	1	1	1	1	2	2
350	ENTECAVIR	3	4	1	1	1	1	2	1	1	1	1	1	1	1	2	1
351	ENZALUTAMIDE	1	1	1	1	1	1	2	1	3	2	1	1	1	1	2	1
352	EPHEDRINE	1	1	1	1	1	1	1	1	1	1	1	1	1	1	1	1
353	EPINASTINE	2	3	2	1	3	3	2	1	1	1	1	1	2	1	2	2
354	EPINEPHRINE	1	2	1	1	1	1	1	1	1	1	1	1	1	1	1	1
355	EPIRUBICIN	1	1	1	1	1	1	1	1	1	1	1	1	1	1	1	1
356	EPLERENONE	1	2	1	1	1	1	2	1	1	2	1	1	1	1	1	1
357	EPOPROSTENOL	1	2	1	1	1	1	1	1	1	1	1	1	1	1	1	1
358	EPROSARTAN	1	1	1	1	1	1	2	1	1	1	1	1	1	1	1	1
359	ERAVACYCLINE	1	1	1	1	1	1	1	2	1	1	1	1	1	1	1	1
360	ERGOCALCIFEROL	1	1	1	1	1	1	2	2	2	2	1	1	1	1	2	1
361	ERGOTAMINE	1	1	1	3	1	1	1	4	1	1	1	1	1	1	1	1
362	ERLOTINIB	1	2	1	1	1	1	2	1	1	1	1	1	1	1	1	2
363	ERTAPENEM	1	2	1	1	1	1	2	1	1	1	1	1	1	1	1	1
364	ERTUGLIFLOZIN	1	2	1	1	1	1	2	1	1	1	1	1	1	1	1	1
365	ESCITALOPRAM	1	2	1	1	1	1	2	1	1	1	1	1	1	1	2	2
366	ESLICARBAZEPINE	2	3	4	1	3	3	2	1	1	1	1	1	1	1	2	2
367	ESMOLOL	1	2	1	1	1	1	2	1	1	1	1	1	1	1	2	1
368	ESOMEPRAZOLE	1	2	1	1	1	1	2	1	1	1	1	1	1	1	2	2
369	ESTAZOLAM	1	3	3	2	2	4	2	2	1	1	1	1	1	1	2	2
370	ESTRADIOL	4	4	4	3	4	4	2	1	2	2	2	1	1	1	3	2

No.	Name	AR	ARan.	ERα	ERαan.	ERβ	ERβan.	GR	GRan.	LXRα	LXRβ	PPARα	PPARβ	PPARγ	RXRα	TRα	TRβ
371	ESTRAMUSTINE	1	1	1	1	1	1	2	2	2	2	1	1	1	1	1	2
372	ESTRONE	4	4	4	3	4	4	3	2	2	2	1	1	1	2	3	3
373	ESZOPICLONE	1	2	1	1	1	1	2	1	1	1	1	1	1	1	1	2
374	ETHACRYNIC ACID	2	2	1	1	1	1	1	1	1	1	1	1	1	1	2	1
375	ETHAMBUTOL	1	2	1	1	1	1	1	1	1	1	1	1	1	1	1	1
376	ETHANOLAMINE	1	2	1	1	1	1	1	1	1	1	1	1	1	1	1	1
377	ETHINYL ESTRADIOL	3	4	4	2	3	1	2	1	2	2	1	1	1	1	1	3
378	ETHIONAMIDE	1	2	1	1	1	1	1	1	1	1	1	1	1	1	1	1
379	ETHOSUXIMIDE	1	2	1	1	1	1	1	1	1	1	1	1	1	1	1	1
380	ETHOTOIN	1	3	1	1	1	1	1	1	1	1	1	1	1	1	2	1
381	ETHYNODIOL	4	4	4	3	1	1	2	1	1	2	1	1	1	1	1	2
382	ETODOLAC	1	3	1	1	2	1	1	1	1	1	1	1	1	1	1	2
383	ETOMIDATE	1	2	1	1	1	1	1	1	1	1	1	1	1	1	2	1
384	ETONOGESTREL	2	4	2	1	3	1	2	1	2	2	1	1	1	1	1	2
385	ETOPOSIDE	1	1	1	1	1	1	2	1	1	1	1	1	2	1	1	1
386	ETRAVIRINE	1	1	1	1	1	1	2	2	1	1	1	1	1	1	1	1
387	EXEMESTANE	2	3	1	2	1	1	2	2	2	2	1	1	1	1	1	2
388	EZETIMIBE	1	1	4	4	1	4	2	2	1	2	2	1	4	2	1	2
389	FAMOTIDINE	1	2	1	1	1	1	1	1	1	1	1	1	1	1	1	1
390	FEBUXOSTAT	1	1	1	1	1	1	2	1	1	1	1	1	1	1	2	2
391	FELBAMATE	1	2	1	1	1	1	1	1	1	1	1	1	1	1	1	1
392	FELODIPINE	1	2	1	1	1	1	2	1	1	1	1	1	1	1	1	1
393	FENOFIBRATE	1	2	1	1	1	1	2	1	1	1	1	1	1	1	2	2
394	FENOFIBRIC ACID	1	2	1	1	1	1	2	1	1	1	1	1	1	1	2	2
395	FENOLDOPAM	3	4	1	1	1	1	2	1	1	1	1	1	1	1	2	2
396	FENOPROFEN	2	3	1	1	2	1	2	1	1	1	1	1	1	1	2	2
397	FENTANYL	1	4	2	1	1	2	2	1	1	1	1	1	1	1	2	2
398	FESOTERODINE	1	1	1	1	1	1	2	2	1	1	1	1	1	1	1	1
399	FEXOFENADINE	1	1	4	3	1	3	2	2	2	2	3	2	2	1	1	1

No.	Name	AR	ARan.	ERα	ERαan.	ERβ	ERβan.	GR	GRan.	LXRα	LXRβ	PPARα	PPARβ	PPARγ	RXRα	TRα	TRβ
400	FINASTERIDE	1	2	1	1	1	1	2	2	2	3	1	1	1	1	2	3
401	FINGOLIMOD	1	2	1	1	1	1	1	1	1	1	1	1	1	1	2	2
402	FLAVIN MONONUCLEOTIDE	1	2	1	1	1	1	2	1	1	1	1	1	1	1	1	1
403	FLAVOXATE	1	2	4	1	1	2	2	2	1	1	1	1	2	3	1	1
404	FLECAINIDE	1	3	2	1	1	3	2	1	1	1	1	1	1	1	2	2
405	FLIBANSERIN	1	2	4	2	2	2	3	2	2	2	2	2	4	2	3	3
406	FLORBETABEN F-18	1	2	1	1	1	1	2	1	1	1	1	1	1	1	2	1
407	FLOXURIDINE	1	2	1	1	1	1	1	1	1	1	1	1	1	1	2	1
408	FLUCICLOVINE F-18	1	2	1	1	1	1	1	1	1	1	1	1	1	1	1	1
409	FLUCONAZOLE	2	3	1	1	1	1	2	1	1	1	1	1	1	1	2	1
410	FLUCYTOSINE	1	2	1	1	1	1	1	1	1	1	1	1	1	1	1	1
411	FLUDARABINE	2	2	1	1	1	1	2	1	1	1	1	1	1	1	1	1
412	FLUDEOXYGLUCOSE F-18	1	2	1	1	1	1	1	1	1	1	1	1	1	1	1	1
413	FLUDROCORTISONE	2	3	2	2	1	1	2	1	3	1	1	1	1	1	1	1
414	FLUMAZENIL	3	4	1	1	2	1	2	1	1	1	1	1	1	1	1	2
415	FLUNISOLIDE	1	1	1	1	1	1	1	2	1	1	1	1	1	1	1	1
416	FLUOCINOLONE ACETONIDE	1	1	1	1	1	1	3	3	1	4	1	1	1	1	1	1
417	FLUOCINONIDE	1	1	1	1	1	1	3	1	1	1	1	1	1	1	1	1
418	FLUORESCEIN	1	1	2	2	1	4	2	2	1	1	2	1	1	3	1	1
419	FLUOROMETHOLONE	4	4	1	1	1	1	2	1	1	1	1	1	1	1	1	1
420	FLUOROURACIL	1	1	1	1	1	1	1	1	1	1	1	1	1	1	1	1
421	FLUOXETINE	3	4	2	1	2	1	2	1	1	1	1	1	1	1	2	2
422	FLUOXYMESTERONE	4	4	1	1	2	1	2	1	3	2	1	1	1	1	1	1
423	FLUPHENAZINE	1	2	1	2	1	1	2	2	1	1	1	1	1	1	1	1
424	FLURANDRENOLIDE	1	1	1	1	1	1	2	2	1	2	1	1	1	1	1	1
425	FLURAZEPAM	1	1	1	1	1	1	2	2	1	1	1	1	1	1	2	1
426	FLURBIPROFEN	3	3	1	1	1	1	2	1	1	1	1	1	1	1	2	3
427	FLUTAMIDE	2	3	1	1	2	1	2	1	1	1	1	1	1	1	2	2
428	FLUTICASONE FUROATE	1	1	1	1	1	1	2	2	1	2	1	1	1	1	1	1

No.	Name	AR	ARan.	ERα	ERαan.	ERβ	ERβan.	GR	GRan.	LXRα	LXRβ	PPARα	PPARβ	PPARγ	RXRα	TRα	TRβ
429	FLUTICASONE PROPIONATE	1	1	1	1	1	1	1	2	1	1	1	1	1	1	1	1
430	FLUVASTATIN	1	2	1	1	1	2	1	1	1	1	1	1	1	1	1	1
431	FLUVOXAMINE	1	2	1	1	1	1	1	1	1	1	1	1	1	1	2	1
432	FOLIC ACID	1	2	1	1	1	1	2	1	1	1	1	1	1	1	2	2
433	FOMEPIZOLE	1	2	1	1	1	1	1	1	1	1	1	1	1	1	1	1
434	FORMOTEROL	1	2	1	1	1	1	2	1	1	1	1	1	1	1	2	2
435	FOSCARNET	1	2	1	1	1	1	1	1	1	1	1	1	1	1	1	1
436	FOSFOMYCIN	1	2	1	1	1	1	1	1	1	1	1	1	1	1	1	1
437	FOSINOPRILAT	1	1	1	1	1	1	2	1	1	1	1	1	1	1	1	1
438	FOSTAMATINIB	1	1	1	1	1	1	1	1	1	1	1	1	1	1	1	1
439	FROVATRIPTAN	2	3	1	1	1	1	2	1	1	1	1	1	1	1	2	1
440	FUROSEMIDE	2	1	1	1	1	1	2	1	1	1	1	1	1	1	2	1
441	GABAPENTIN	1	2	1	1	1	1	1	1	1	1	1	1	1	1	1	1
442	GADODIAMIDE	1	1	1	1	1	1	1	1	1	1	1	1	1	1	1	1
443	GALANTAMINE	1	3	2	1	2	1	2	1	1	1	1	1	1	1	1	2
444	GANCICLOVIR	1	2	1	1	1	1	1	1	1	1	1	1	1	1	2	1
445	GATIFLOXACIN	1	2	1	1	1	1	2	1	1	1	1	1	1	1	1	1
446	GEFITINIB	1	1	1	1	1	1	2	2	1	1	1	1	1	1	1	1
447	GEMCITABINE	2	2	1	1	1	1	1	1	1	1	1	1	1	1	1	1
448	GEMFIBROZIL	2	2	1	1	1	1	1	1	1	1	1	1	1	1	2	1
449	GEMIFLOXACIN	1	2	1	1	1	1	2	1	1	1	1	1	1	1	1	2
450	GILTERITINIB	1	1	1	1	1	3	1	2	1	1	1	1	2	1	1	1
451	GLASDEGIB	1	2	2	1	1	1	3	2	1	1	1	1	1	1	1	1
452	GLIMEPIRIDE	1	1	4	4	1	3	2	3	3	2	4	1	3	2	1	1
453	GLIPIZIDE	1	1	4	4	1	3	3	2	2	1	3	1	2	1	1	2
454	GLUCONIC ACID	1	2	1	1	1	1	1	1	1	1	1	1	1	1	1	1
455	GLUCONOLACTONE	1	2	1	1	1	1	1	1	1	1	1	1	1	1	1	1
456	GLUTAMINE	1	1	1	1	1	1	1	1	1	1	1	1	1	1	1	1
457	GLYBURIDE/GLIBENCLAMIDE	1	2	4	3	1	3	3	3	2	2	2	1	2	1	1	1

No.	Name	AR	ARan.	ERα	ERαan.	ERβ	ERβan.	GR	GRan.	LXRα	LXRβ	PPARα	PPARβ	PPARγ	RXRα	TRα	TRβ
458	GLYCERIN	1	2	1	1	1	1	1	1	1	1	1	1	1	1	1	1
459	GLYCINE	1	2	1	1	1	1	1	1	1	1	1	1	1	1	1	1
460	GLYCOPYRRONIUM	2	3	1	2	2	1	2	1	1	1	1	1	1	1	2	2
461	GRANISETRON	2	4	2	3	1	1	2	2	1	1	1	1	1	1	2	2
462	GRISEOFULVIN	1	2	1	1	1	1	2	1	1	1	1	1	1	1	1	1
463	GUAIFENESIN	1	1	1	1	1	1	1	1	1	1	1	1	1	1	1	1
464	GUANFACINE	2	2	1	1	1	1	1	1	1	1	1	1	1	1	1	1
465	HALCINONIDE	1	1	1	1	1	1	3	3	1	4	1	1	1	1	1	1
466	HALOBETASOL	1	2	1	1	1	1	2	1	1	2	1	1	1	1	1	1
467	HALOPERIDOL	1	3	3	3	2	2	2	2	2	2	1	1	1	2	2	1
468	HALOPROGIN	1	2	1	1	1	1	1	1	1	1	1	1	1	1	1	1
469	HEXACHLOROPHENE	1	3	1	1	1	1	2	1	1	1	1	1	1	1	2	1
470	HEXAMINOLEVULINATE	1	1	1	1	1	1	1	1	1	1	1	1	1	1	1	1
471	HYDRALAZINE	1	2	1	1	1	1	1	1	1	1	1	1	1	1	1	1
472	HYDROCHLOROTHIAZIDE	1	1	1	1	1	1	1	1	1	1	1	1	1	1	1	1
473	HYDROCODONE	1	4	2	1	1	2	2	1	2	1	1	1	1	1	1	2
474	HYDROCORTISONE	1	2	1	1	1	1	2	1	1	2	1	1	1	1	1	2
475	HYDROMORPHONE	1	4	4	2	4	4	2	2	2	1	1	1	1	1	2	3
476	HYDROQUINONE	1	2	1	1	1	1	1	1	1	1	1	1	1	1	1	1
477	HYDROXYAMPHETAMINE	1	2	1	1	1	1	1	1	1	1	1	1	1	1	1	1
478	HYDROXYCHLOROQUINE	1	2	1	1	1	1	2	1	1	1	1	1	1	1	2	2
479	HYDROXYPROGESTERONE	2	3	1	1	1	1	3	1	1	3	1	1	1	1	1	2
480	HYDROXYUREA	1	1	1	1	1	1	1	1	1	1	1	1	1	1	1	1
481	HYDROXYZINE	1	2	1	1	1	1	1	1	1	1	1	1	1	1	1	1
482	IBANDRONIC ACID	1	1	1	1	1	1	1	1	1	1	1	1	1	1	1	1
483	IBRUTINIB	1	1	1	2	1	3	3	4	3	4	2	4	4	1	1	1
484	IBUPROFEN	1	1	1	1	1	1	1	1	1	1	1	1	1	1	2	1
485	IBUTILIDE	1	2	1	1	1	1	2	1	1	1	1	1	1	1	2	1
486	ICOSAPENT	1	2	1	1	1	1	1	1	1	1	1	1	1	1	2	1

No.	Name	AR	ARan.	ERα	ERαan.	ERβ	ERβan.	GR	GRan.	LXRα	LXRβ	PPARα	PPARβ	PPARγ	RXRα	TRα	TRβ
487	IDARUBICIN	1	1	1	1	1	1	1	2	1	2	1	1	2	1	1	1
488	IDELALISIB	1	2	1	1	1	1	2	2	3	2	2	1	3	1	1	2
489	ILOPERIDONE	1	2	2	2	1	3	2	1	2	2	2	3	2	1	1	1
490	IMATINIB	1	1	1	4	1	3	3	3	3	2	3	1	2	1	1	1
491	IMIPENEM	1	1	1	1	1	1	1	1	1	1	1	1	1	1	1	1
492	IMIPRAMINE	1	3	3	1	2	2	2	1	1	1	1	1	1	1	2	2
493	IMIQUIMOD	1	2	1	1	1	1	2	1	1	1	1	1	1	1	2	2
494	INAMRINONE	1	2	1	1	1	1	2	1	1	1	1	1	1	1	2	1
495	INDACATEROL	1	1	2	1	1	2	3	2	2	3	1	1	2	1	2	2
496	INDAPAMIDE	1	1	3	4	1	1	2	2	1	1	1	1	1	1	2	2
497	INDOMETHACIN	1	1	1	1	1	1	2	2	1	1	1	1	1	1	1	1
498	INGENOL MEBUTATE	1	2	1	1	1	1	1	1	1	1	1	1	1	1	1	1
499	IOBENGUANE I-123	1	2	1	1	1	1	1	1	1	1	1	1	1	1	2	1
500	IOBENGUANE I-131	1	2	1	1	1	1	1	1	1	1	1	1	1	1	2	1
501	IODIDE ION	1	1	1	1	1	1	1	1	1	1	1	1	1	1	1	1
502	IODIDE ION I-123	1	1	1	1	1	1	1	1	1	1	1	1	1	1	1	1
503	IODIDE ION I-125	1	1	1	1	1	1	1	1	1	1	1	1	1	1	1	1
504	IODINE	1	1	1	1	1	1	1	1	1	1	1	1	1	1	1	1
505	IOFLUPANE I-123	1	2	1	1	1	1	1	1	1	1	1	1	1	1	1	1
506	IPRATROPIUM	1	3	1	1	1	1	2	1	1	1	1	1	1	1	2	2
507	IRBESARTAN	1	1	1	4	1	4	2	2	2	3	1	4	3	1	1	2
508	IRINOTECAN	1	1	1	4	1	4	1	4	1	1	2	3	2	1	1	1
509	ISAVUCONAZOLE	1	2	1	3	1	1	3	2	2	2	1	1	1	1	2	3
510	ISOCARBOXAZID	1	2	1	1	1	1	2	1	1	1	1	1	1	1	2	1
511	ISOFLURANE	1	2	1	1	1	1	1	1	1	1	1	1	1	1	1	1
512	ISONIAZID	1	2	1	1	1	1	1	1	1	1	1	1	1	1	1	1
513	ISOPROPYL ALCOHOL	1	2	1	1	1	1	1	1	1	1	1	1	1	1	1	1
514	ISOPROTERENOL	1	2	1	1	1	1	1	1	1	1	1	1	1	1	1	1
515	ISOSORBIDE DINITRATE	1	2	1	1	1	1	1	1	1	1	1	1	1	1	1	1

No.	Name	AR	ARan.	ERα	ERαan.	ERβ	ERβan.	GR	GRan.	LXRα	LXRβ	PPARα	PPARβ	PPARγ	RXRα	TRα	TRβ
516	ISOSORBIDE MONONITRATE	1	2	1	1	1	1	1	1	1	1	1	1	1	1	1	1
517	ISOSULFAN BLUE INNER SALT	1	1	1	1	1	1	1	1	1	1	1	1	1	1	1	1
518	ISRADIPINE	1	2	1	1	1	3	1	1	1	1	1	1	1	1	1	1
519	ISTRADEFYLLINE	1	1	1	1	1	1	2	1	1	1	1	1	1	1	1	1
520	IVABRADINE	1	2	1	1	1	1	2	1	1	1	1	1	2	1	1	1
521	IVACAFTOR	1	2	1	1	1	2	2	1	1	1	1	3	1	1	1	1
522	IVOSIDENIB	1	1	1	1	1	1	1	1	1	1	1	1	1	1	1	1
523	IXABEPILONE	1	1	1	1	1	1	1	1	1	1	1	1	1	1	1	1
524	KETAMINE	1	1	1	1	1	1	1	1	1	1	1	1	1	1	1	2
525	KETOCONAZOLE	1	1	4	4	1	4	2	2	3	1	2	1	1	1	1	1
526	KETOPROFEN	3	3	2	1	2	2	2	2	1	1	1	1	1	1	3	2
527	KETOROLAC	3	4	1	1	2	1	2	1	1	1	1	1	1	1	2	2
528	KETOTIFEN	1	1	3	2	1	3	2	2	1	1	1	1	1	1	1	1
529	LABETALOL	1	3	2	1	2	1	2	1	1	1	1	1	1	1	3	2
530	LACOSAMIDE	1	1	1	1	1	1	1	1	1	1	1	1	1	1	2	1
531	LACTIC ACID, DL-	1	2	1	1	1	1	1	1	1	1	1	1	1	1	1	1
532	LAMIVUDINE	1	1	1	1	1	1	1	1	1	1	1	1	1	1	1	1
533	LAMOTRIGINE	2	2	1	1	1	1	1	1	1	1	1	1	1	1	2	1
534	LANSOPRAZOLE	1	2	2	1	2	2	2	2	1	1	1	1	1	1	3	3
535	LAPATINIB	1	1	2	3	1	2	2	2	2	1	4	3	4	2	1	1
536	LAROTRECTINIB	1	2	2	1	1	4	2	3	3	4	1	2	1	1	1	1
537	LATANOPROST	1	1	2	1	1	1	2	2	1	1	1	1	1	1	2	2
538	LATANOPROST	1	3	2	1	1	1	2	1	1	1	1	1	1	1	2	2
539	LENALIDOMIDE	2	3	2	2	2	1	2	2	1	1	1	1	1	1	2	2
540	LENVATINIB	1	2	1	1	1	1	3	2	2	2	1	1	1	1	1	2
541	LETERMOVIR	1	1	1	1	1	1	1	1	1	1	1	1	2	1	1	1
542	LETROZOLE	1	1	1	1	1	1	1	1	1	1	1	1	1	1	1	1
543	LEUCOVORIN	1	2	1	1	1	1	2	1	1	1	1	1	1	1	1	1
544	LEVALBUTEROL	1	2	1	1	1	1	1	1	1	1	1	1	1	1	2	1

No.	Name	AR	ARan.	ERα	ERαan.	ERβ	ERβan.	GR	GRan.	LXRα	LXRβ	PPARα	PPARβ	PPARγ	RXRα	TRα	TRβ
545	LEVETIRACETAM	1	1	1	1	1	1	1	1	1	1	1	1	1	1	1	1
546	LEVOBUNOLOL	2	3	1	1	1	1	2	1	1	1	1	1	1	1	2	2
547	LEVOCARNITINE	1	2	1	1	1	1	1	1	1	1	1	1	1	1	1	1
548	LEVOCETIRIZINE	1	1	1	1	1	1	2	2	1	1	1	1	1	1	2	2
549	LEVODOPA	1	1	1	1	1	1	1	1	1	1	1	1	1	1	1	1
550	LEVOFLOXACIN	1	1	1	1	1	1	2	2	1	1	1	1	1	1	2	2
551	LEVOLEUCOVORIN	1	2	1	1	1	1	2	2	1	1	1	1	1	1	1	1
552	LEVOMENTHOL	1	2	1	1	1	1	1	1	1	1	1	1	1	1	1	1
553	LEVOMILNACIPRAN	1	2	1	1	1	1	1	1	1	1	1	1	1	1	2	1
554	LEVONORDEFRIN	1	2	1	1	1	1	1	1	1	1	1	1	1	1	1	1
555	LEVONORGESTREL	2	3	2	1	3	1	2	1	2	2	1	1	1	1	1	2
556	LEVORPHANOL	4	4	4	2	4	4	2	1	1	2	1	1	1	1	1	2
557	LIDOCAINE	1	1	1	1	1	1	1	1	1	1	1	1	1	1	1	1
558	LINAGLIPTIN	1	1	1	1	1	1	2	2	1	2	1	1	1	1	1	1
559	LINCOMYCIN	1	1	1	1	1	1	2	1	1	1	1	1	1	1	1	1
560	LINDANE	1	2	1	1	1	1	1	1	1	1	1	1	1	1	1	1
561	LINEZOLID	1	1	1	1	1	1	2	2	1	1	1	1	1	1	2	2
562	LISDEXAMFETAMINE	1	2	1	1	1	1	1	1	1	1	1	1	1	1	2	2
563	LISINOPRIL	1	1	1	1	1	2	2	1	1	1	1	1	1	1	2	2
564	LODOXAMIDE	1	1	1	1	1	1	2	1	1	1	1	1	1	1	1	1
565	LOFEXIDINE	1	2	1	1	1	1	1	1	1	1	1	1	1	1	1	1
566	LOMUSTINE	1	2	1	1	1	1	1	1	1	1	1	1	1	1	1	1
567	LOPERAMIDE	1	1	1	1	1	1	2	3	1	1	1	1	1	1	1	2
568	LORATADINE	1	1	1	1	1	3	1	3	1	1	1	1	1	1	1	1
569	LORAZEPAM	1	3	3	2	4	3	2	2	1	1	1	1	1	1	1	2
570	LORCASERIN	1	2	1	1	1	1	1	1	1	1	1	1	1	1	1	1
571	LORLATINIB	1	1	1	1	1	1	1	1	1	1	1	1	1	1	1	1
572	LOSARTAN	1	1	1	3	1	2	2	2	1	2	1	2	3	1	1	2
573	LOTEPREDNOL	1	2	1	1	1	1	2	1	1	2	1	1	1	1	1	3

No.	Name	AR	ARan.	ERα	ERαan.	ERβ	ERβan.	GR	GRan.	LXRα	LXRβ	PPARα	PPARβ	PPARγ	RXRα	TRα	TRβ
574	LOVASTATIN ACID	1	1	1	1	1	1	2	2	1	1	1	1	1	2	1	2
575	LOXAPINE	1	2	2	1	1	2	1	2	2	1	1	1	1	1	1	1
576	LUBIPROSTONE	1	2	1	1	1	1	2	1	1	1	1	1	1	1	2	2
577	LULICONAZOLE	1	2	1	1	1	1	2	1	1	1	1	1	1	1	1	1
578	LUMACAFTOR	1	2	2	2	1	4	3	4	3	4	3	4	3	3	1	2
579	LUMEFANTRINE	1	1	1	1	1	1	2	1	1	1	1	1	1	1	1	1
580	LURASIDONE	1	1	1	3	1	4	2	4	4	4	1	3	2	1	1	1
581	LUSUTROMBOPAG	1	1	1	1	1	1	1	1	1	1	1	1	1	1	1	1
582	MACIMORELIN	1	2	3	2	1	3	3	3	3	3	2	3	3	2	2	3
583	MACITENTAN	1	2	2	1	1	1	2	1	1	1	1	1	1	1	1	1
584	MAFENIDE	1	2	1	1	1	1	1	1	1	1	1	1	1	1	1	1
585	MALATHION	1	2	1	1	1	1	1	1	1	1	1	1	1	1	1	1
586	MAPROTILINE	2	3	4	3	3	2	2	2	1	1	1	1	1	1	2	2
587	MARAVIROC	1	1	1	4	1	4	2	2	3	3	4	4	3	1	2	1
588	MEBENDAZOLE	1	2	1	2	1	2	2	1	2	1	2	1	1	1	2	3
589	MECAMYLAMINE	1	2	1	1	1	1	1	1	1	1	1	1	1	1	1	1
590	MECHLORETHAMINE	1	2	1	1	1	1	1	1	1	1	1	1	1	1	1	1
591	MECLIZINE	1	2	3	2	1	4	2	3	3	2	1	1	3	1	1	2
592	MECLOFENAMIC ACID	3	4	2	2	2	1	2	1	1	1	1	1	1	1	2	2
593	MEDROXYPROGESTERONE	4	4	1	1	1	1	2	1	2	1	1	1	1	1	1	1
594	MEFENAMIC ACID	2	3	3	2	2	1	2	1	1	1	1	1	1	1	2	2
595	MEFLOQUINE	2	4	3	1	3	4	2	2	2	1	1	1	1	1	4	3
596	MEGESTROL	4	3	1	1	1	1	2	1	1	3	1	1	1	1	1	2
597	MELOXICAM	2	3	1	1	1	1	2	2	1	1	1	1	1	1	1	1
598	MELPHALAN	1	1	1	1	1	1	1	1	1	1	1	1	1	1	1	1
599	MEMANTINE	1	1	1	1	1	1	1	1	1	1	1	1	1	1	1	1
600	MEPERIDINE	1	2	1	1	1	1	1	1	1	1	1	1	1	1	2	2
601	MEPIVACAINE	2	2	2	1	1	2	2	2	1	1	1	1	1	1	2	2
602	MEPROBAMATE	1	2	1	1	1	1	1	1	1	1	1	1	1	1	1	1

No.	Name	AR	ARan.	ERα	ERαan.	ERβ	ERβan.	GR	GRan.	LXRα	LXRβ	PPARα	PPARβ	PPARγ	RXRα	TRα	TRβ
603	MERCAPTOPURINE	1	1	1	1	1	1	1	1	1	1	1	1	1	1	1	1
604	MEROPENEM	1	1	1	1	1	1	2	1	1	1	1	1	1	1	1	1
605	MESALAMINE	1	2	1	1	1	1	1	1	1	1	1	1	1	1	1	1
606	METAPROTERENOL	1	2	1	1	1	1	1	1	1	1	1	1	1	1	1	1
607	METAXALONE	1	2	1	1	1	1	1	1	1	1	1	1	1	1	2	2
608	METFORMIN	1	1	1	1	1	1	1	1	1	1	1	1	1	1	1	1
609	METHACHOLINE	1	2	1	1	1	1	1	1	1	1	1	1	1	1	1	1
610	METHADONE	1	1	1	2	1	2	2	2	1	1	1	1	1	1	2	2
611	METHAMPHETAMINE	1	2	1	1	1	1	1	1	1	1	1	1	1	1	1	1
612	METHAZOLAMIDE	1	2	1	1	1	1	1	1	1	1	1	1	1	1	1	1
613	METHENAMINE	1	2	1	1	1	1	1	1	1	1	1	1	1	1	1	1
614	METHIMAZOLE	1	2	1	1	1	1	1	1	1	1	1	1	1	1	1	1
615	METHOCARBAMOL	1	2	1	1	1	1	1	1	1	1	1	1	1	1	1	1
616	METHOHEXITAL	1	2	1	1	1	1	1	1	1	1	1	1	1	1	1	1
617	METHOTREXATE	1	2	1	1	1	1	2	1	1	1	1	1	1	1	2	2
618	METHOXSALEN	2	3	1	1	1	1	2	1	1	1	1	1	1	1	2	1
619	METHSCOPOLAMINE	1	4	1	1	1	1	2	1	1	1	1	1	1	1	1	2
620	METHYLDOPA	1	2	1	1	1	1	1	1	1	1	1	1	1	1	1	1
621	METHYLENE BLUE CATION	1	2	1	1	1	1	2	1	1	1	1	1	1	1	2	1
622	METHYLERGONOVINE	1	2	1	1	1	1	2	1	1	1	1	1	1	1	2	2
623	METHYLHOMATROPINE	3	3	1	1	1	1	2	1	1	1	1	1	1	1	2	2
624	METHYLNALTREXONE	1	3	3	1	1	3	2	2	2	2	1	1	1	1	1	4
625	METHYLPHENIDATE	1	1	1	1	1	1	1	1	1	1	1	1	1	1	2	2
626	METHYLPREDNISOLONE	1	2	1	1	1	1	2	1	1	1	1	1	1	1	1	2
627	METHYLTESTOSTERONE	4	4	1	2	2	1	2	1	1	3	1	1	1	1	2	3
628	METOCLOPRAMIDE	1	1	1	1	1	1	1	1	1	1	1	1	1	1	1	1
629	METOLAZONE	1	3	1	1	1	1	2	1	1	1	1	1	1	1	1	2
630	METOPROLOL	1	1	1	1	1	1	1	1	1	1	1	1	1	1	2	1
631	METRONIDAZOLE	1	2	1	1	1	1	1	1	1	1	1	1	1	1	1	1

No.	Name	AR	ARan.	ERα	ERαan.	ERβ	ERβan.	GR	GRan.	LXRα	LXRβ	PPARα	PPARβ	PPARγ	RXRα	TRα	TRβ
632	METYROSINE	1	2	1	1	1	1	1	1	1	1	1	1	1	1	1	1
633	MEXILETINE	1	2	1	1	1	1	1	1	1	1	1	1	1	1	1	1
634	MICONAZOLE	1	2	2	1	1	1	2	1	1	1	1	1	1	1	1	2
635	MIDAZOLAM	1	2	2	1	2	2	2	2	1	1	1	1	1	1	2	1
636	MIDODRINE	1	2	1	1	1	1	1	1	1	1	1	1	1	1	1	1
637	MIDOSTAURIN	1	1	1	2	1	1	1	3	1	1	1	1	1	1	1	1
638	MIFEPRISTONE	1	1	1	1	1	1	1	1	1	1	1	1	2	1	1	1
639	MIGALASTAT	1	2	1	1	1	1	1	1	1	1	1	1	1	1	1	1
640	MIGLITOL	1	2	1	1	1	1	1	1	1	1	1	1	1	1	1	1
641	MIGLUSTAT	1	1	1	1	1	1	1	1	1	1	1	1	1	1	1	1
642	MILNACIPRAN	1	2	1	1	1	1	1	1	1	1	1	1	1	1	2	1
643	MILRINONE	1	2	1	1	1	1	2	1	1	1	1	1	1	1	2	1
644	MILTEFOSINE	1	2	1	1	1	1	1	1	1	1	1	1	1	1	1	1
645	MINOCYCLINE	1	1	1	1	1	1	1	1	1	1	1	1	1	1	1	1
646	MINOXIDIL	1	2	1	1	1	1	1	1	1	1	1	1	1	1	2	1
647	MIRABEGRON	1	2	1	1	1	2	2	1	1	1	2	1	2	1	2	2
648	MIRTAZAPINE	2	3	2	1	3	1	2	2	1	1	1	1	1	1	3	2
649	MISOPROSTOL	1	2	1	1	1	1	2	1	1	1	1	1	1	1	1	1
650	MITOMYCIN	1	3	2	1	1	1	2	1	1	1	1	1	1	1	1	1
651	MITOTANE	2	2	2	2	2	2	2	2	1	1	1	1	1	1	2	2
652	MITOXANTRONE	1	2	1	1	1	1	1	1	1	1	1	1	1	1	1	1
653	MOEXIPRILAT	1	2	1	1	1	1	2	1	2	1	1	1	1	1	1	1
654	MOLINDONE	1	3	1	1	1	1	2	1	1	1	1	1	1	1	2	2
655	MOMETASONE	1	2	1	1	1	1	2	2	1	1	1	1	1	1	1	2
656	MONOFLUOROPHOSPHATE ION	1	2	1	1	1	1	1	1	1	1	1	1	1	1	1	1
657	MONOMETHYL FUMARATE	1	2	1	1	1	1	1	1	1	1	1	1	1	1	1	1
658	MONTELUKAST	1	1	3	4	1	4	2	3	2	2	2	3	4	1	1	1
659	MORPHINE	1	3	4	2	4	3	2	2	1	1	1	1	1	1	1	2
660	MOXIFLOXACIN	1	1	1	1	1	1	2	1	1	1	1	1	1	1	1	1

No.	Name	AR	ARan.	ERα	ERαan.	ERβ	ERβan.	GR	GRan.	LXRα	LXRβ	PPARα	PPARβ	PPARγ	RXRα	TRα	TRβ
661	MUPIROCIN	1	1	1	1	1	1	2	2	1	1	1	1	1	1	1	1
662	MYCOPHENOLIC ACID	2	1	1	1	1	1	2	2	1	1	1	1	1	1	2	2
663	NABILONE	1	2	1	1	1	2	2	1	1	2	1	1	1	1	1	2
664	NABUMETONE	2	3	1	1	1	1	2	1	1	1	1	1	1	1	2	2
665	NAFCILLIN	1	2	1	1	1	1	2	1	1	1	1	1	1	1	1	1
666	NAFTIFINE	2	4	3	2	4	3	2	2	2	2	1	2	2	1	3	2
667	NALBUPHINE	1	2	1	1	1	1	2	2	1	1	1	1	1	1	1	1
668	NALDEMEDINE	1	1	1	2	1	4	1	4	1	1	1	1	1	1	1	1
669	NALOXONE	1	2	1	1	1	1	2	1	1	1	1	1	1	1	1	1
670	NALTREXONE	2	4	1	2	1	1	2	2	1	1	1	1	1	1	1	2
671	NAPHAZOLINE	2	3	1	1	2	1	2	2	1	1	1	1	1	1	2	2
672	NAPROXEN	3	3	1	1	1	1	2	1	1	1	1	1	1	1	2	2
673	NARATRIPTAN	1	2	2	1	3	1	2	2	1	1	1	1	1	1	1	2
674	NATEGLINIDE	2	3	1	1	1	1	2	1	1	1	1	1	1	1	2	2
675	NBI-98782	1	2	1	1	1	1	2	1	1	1	1	1	1	1	1	2
676	NEBIVOLOL	3	4	4	4	1	3	2	2	2	2	3	3	3	2	2	1
677	NEDOCROMIL	1	2	1	1	1	1	2	1	1	1	1	1	1	1	1	1
678	NEFAZODONE	1	2	2	2	1	2	2	2	2	1	2	2	3	1	1	1
679	NELARABINE	1	3	1	1	1	1	1	1	1	1	1	1	1	1	1	1
680	NELFINAVIR	1	1	1	4	1	4	2	4	1	1	4	4	4	1	1	1
681	NEOSTIGMINE	1	2	1	1	1	1	1	1	1	1	1	1	1	1	1	1
682	NEPAFENAC	2	3	2	1	2	1	2	1	1	1	1	1	1	1	2	2
683	NERATINIB	1	1	1	1	1	1	1	2	1	1	3	1	2	1	1	1
684	NETUPITANT	1	1	1	1	1	3	1	4	1	1	2	4	3	1	1	1
685	NEVIRAPINE	1	1	2	1	1	1	2	2	1	1	1	1	1	1	2	2
686	NIACIN	1	2	1	1	1	1	1	1	1	1	1	1	1	1	1	1
687	NIACINAMIDE	1	2	1	1	1	1	1	1	1	1	1	1	1	1	1	1
688	NICARDIPINE	1	2	1	1	1	1	2	1	1	1	1	1	1	1	1	1
689	NICOTINE	1	1	1	1	1	1	1	1	1	1	1	1	1	1	1	1

No.	Name	AR	ARan.	ERα	ERαan.	ERβ	ERβan.	GR	GRan.	LXRα	LXRβ	PPARα	PPARβ	PPARγ	RXRα	TRα	TRβ
690	NIFEDIPINE	1	1	2	1	1	1	1	1	1	1	1	1	1	1	1	1
691	NILUTAMIDE	3	4	2	2	1	1	2	1	1	1	1	1	1	1	2	2
692	NIMODIPINE	1	1	1	1	1	1	1	1	1	1	1	1	1	1	1	1
693	NINTEDANIB	1	1	1	2	1	1	1	4	3	1	1	1	2	1	1	1
694	NIRAPARIB	1	1	1	2	1	2	2	2	3	3	1	1	1	1	2	1
695	NISOLDIPINE	1	2	1	1	1	1	1	1	1	1	1	1	1	1	1	1
696	NITISINONE	2	4	2	2	1	2	2	1	1	1	1	1	1	1	2	2
697	NITROFURANTOIN	2	2	1	1	1	1	1	1	1	1	1	1	1	1	2	1
698	NITROGLYCERIN	1	2	1	1	1	1	1	1	1	1	1	1	1	1	1	1
699	NIZATIDINE	1	2	1	1	1	1	1	1	1	1	1	1	1	1	1	1
700	NORELGESTROMIN	2	2	2	1	1	1	2	1	2	2	1	1	1	1	1	2
701	NOREPINEPHRINE	1	2	1	1	1	1	1	1	1	1	1	1	1	1	1	1
702	NORETHINDRONE/NORETHISTERONE	4	4	2	1	3	1	2	1	2	2	1	1	1	1	2	3
703	NORGESTIMATE	1	2	1	1	1	1	2	2	2	1	1	1	1	1	1	2
704	NORTRIPTYLINE	1	3	3	2	2	4	2	1	2	1	1	1	1	1	3	3
705	OBETICHOLIC ACID	1	2	1	1	1	1	1	2	1	1	1	1	1	1	1	1
706	OCTOCRYLENE	1	2	1	1	1	1	2	1	1	1	1	1	1	1	2	2
707	OFLOXACIN	1	2	1	1	1	1	2	1	1	1	1	1	1	1	1	1
708	OLANZAPINE	1	1	1	1	1	1	1	2	1	1	1	1	1	1	1	1
709	OLAPARIB	1	1	1	3	1	3	3	4	3	4	4	4	3	2	1	1
710	OLMESARTAN	1	1	1	2	1	1	2	1	1	1	1	1	2	1	1	1
711	OLODATEROL	1	2	1	1	1	1	2	2	1	1	1	1	2	1	2	2
712	OLOPATADINE	1	4	4	3	1	2	2	2	1	1	1	1	1	1	1	2
713	OLSALAZINE	1	2	1	1	1	1	2	1	1	1	1	1	1	1	2	2
714	OMACETAXINE	1	2	2	2	1	1	2	1	1	1	1	1	1	1	1	1
715	OMADACYCLINE	1	1	1	1	1	1	1	1	1	1	1	1	1	1	1	1
716	OMEPRAZOLE	1	1	1	1	1	1	2	2	1	1	1	1	1	1	2	2
717	ONDANSETRON	2	2	1	2	1	1	2	2	1	1	1	1	1	1	2	2
718	ORLISTAT	1	1	1	1	1	1	2	1	1	1	1	1	1	1	1	1

No.	Name	AR	ARan.	ERα	ERαan.	ERβ	ERβan.	GR	GRan.	LXRα	LXRβ	PPARα	PPARβ	PPARγ	RXRα	TRα	TRβ
719	ORPHENADRINE	1	2	1	1	1	1	2	1	1	1	1	1	1	1	2	2
720	OSELTAMIVIR	1	2	1	1	1	1	1	1	1	1	1	1	1	1	1	1
721	OSIMERTINIB	1	1	1	1	1	1	2	2	1	1	1	1	2	1	1	1
722	OSPEMIFENE	1	2	3	3	1	4	2	1	1	1	1	1	1	1	1	1
723	OXACILLIN	1	2	1	1	1	1	2	2	1	1	1	1	1	1	1	1
724	OXANDROLONE	4	4	1	1	1	4	2	1	2	3	1	1	1	1	2	4
725	OXAPROZIN	1	2	3	2	1	3	2	1	2	1	1	1	2	1	1	2
726	OXAZEPAM	1	3	3	2	4	3	2	2	1	1	1	1	1	1	1	2
727	OXCARBAZEPINE	3	3	4	3	2	3	2	2	1	1	1	1	1	1	2	2
728	OXICONAZOLE	1	2	1	1	1	1	2	1	1	1	1	1	1	1	1	2
729	OXYBUTYNIN	1	1	1	1	1	1	2	1	1	1	1	1	1	1	2	2
730	OXYCODONE	1	3	2	1	2	1	2	1	1	1	1	1	1	1	1	1
731	OXYMETAZOLINE	1	1	1	1	1	1	2	1	1	1	1	1	1	1	2	2
732	OXYMETHOLONE	2	2	1	1	1	1	2	1	1	3	1	1	1	1	1	1
733	OXYMORPHONE	1	4	3	1	4	4	2	2	1	1	1	1	1	1	1	2
734	OXYTETRACYCLINE	1	1	1	1	1	1	1	1	1	1	1	1	1	1	1	1
735	OZENOXACIN	1	1	1	1	1	1	2	1	2	2	1	1	1	1	2	2
736	PALBOCICLIB	1	1	1	2	1	1	1	4	2	1	1	1	1	1	1	1
737	PALIPERIDONE	1	2	2	4	1	2	3	2	3	3	3	4	2	2	2	2
738	PALONOSETRON	4	4	4	3	2	3	2	2	2	2	1	1	1	1	3	3
739	PAMIDRONIC ACID	1	1	1	1	1	1	1	1	1	1	1	1	1	1	1	1
740	PANCURONIUM	1	1	1	1	1	1	1	1	1	1	1	1	1	1	1	1
741	PANOBINOSTAT	1	2	1	2	1	3	2	2	2	1	1	1	2	1	2	3
742	PANTHENOL	1	2	1	1	1	1	1	1	1	1	1	1	1	1	1	1
743	PANTOPRAZOLE	1	1	1	1	1	1	2	2	1	1	1	1	1	1	1	2
744	PARICALCITOL	1	1	1	1	1	1	2	2	3	2	1	1	1	2	2	2
745	PAROXETINE	1	3	4	3	1	3	2	2	2	2	1	1	2	2	2	3
746	PAZOPANIB	1	1	2	2	1	1	2	2	2	2	3	2	3	1	1	1
747	PEMETREXED	1	1	1	2	1	2	2	2	2	2	1	1	1	1	2	2

No.	Name	AR	ARan.	ERα	ERαan.	ERβ	ERβan.	GR	GRan.	LXRα	LXRβ	PPARα	PPARβ	PPARγ	RXRα	TRα	TRβ
748	PENCICLOVIR	1	2	1	1	1	1	1	1	1	1	1	1	1	1	2	1
749	PENICILLAMINE	1	2	1	1	1	1	1	1	1	1	1	1	1	1	1	1
750	PENICILLIN G/BENZYLPENICILLIN	1	4	1	1	1	1	2	1	1	1	1	1	1	1	1	1
751	PENICILLIN V	1	3	1	1	1	1	2	1	1	1	1	1	1	1	1	1
752	PENTAMIDINE	2	3	2	1	1	2	2	1	1	1	1	1	1	1	2	3
753	PENTAZOCINE	1	3	2	1	1	1	2	1	1	1	1	1	1	1	1	2
754	PENTOBARBITAL	1	2	1	1	1	1	1	1	1	1	1	1	1	1	1	1
755	PENTOSTATIN	3	3	1	1	1	1	1	1	1	1	1	1	1	1	2	1
756	PENTOXIFYLLINE	1	1	1	1	1	1	1	1	1	1	1	1	1	1	2	1
757	PERAMIVIR	1	2	1	1	1	1	1	1	1	1	1	1	1	1	1	1
758	PERAMPANEL	1	1	2	3	1	4	1	2	1	1	1	1	2	1	1	1
759	PERFLUTREN	1	2	1	1	1	1	1	1	1	1	1	1	1	1	1	1
760	PERINDOPRIL	1	2	1	1	1	1	2	1	1	1	1	1	1	1	1	2
761	PERINDOPRILAT	1	2	1	1	1	1	2	1	1	1	1	1	1	1	1	1
762	PERMETHRIN	1	1	2	2	2	2	2	2	2	2	1	1	2	2	3	3
763	PERPHENAZINE	1	2	1	1	1	1	2	1	1	1	1	1	1	1	1	1
764	PHENDIMETRAZINE	1	2	1	1	1	1	1	1	1	1	1	1	1	1	1	1
765	PHENELZINE	1	2	1	1	1	1	1	1	1	1	1	1	1	1	1	1
766	PHENINDAMINE	1	2	4	3	4	4	2	2	3	1	2	1	1	1	3	3
767	PHENIRAMINE	1	2	1	1	1	1	1	1	1	1	1	1	1	1	1	1
768	PHENOXYBENZAMINE	1	2	1	1	1	1	1	1	1	1	1	1	1	1	2	2
769	PHENTERMINE	1	2	1	1	1	1	1	1	1	1	1	1	1	1	1	1
770	PHENTOLAMINE	1	3	2	2	1	2	2	1	1	1	1	1	1	1	2	2
771	PHENYLACETIC ACID	1	2	1	1	1	1	1	1	1	1	1	1	1	1	1	1
772	PHENYLBUTYRIC ACID	1	2	1	1	1	1	1	1	1	1	1	1	1	1	1	1
773	PHENYLEPHRINE	1	2	1	1	1	1	1	1	1	1	1	1	1	1	1	1
774	PHENYTOIN	1	3	3	2	3	3	2	1	1	1	1	1	1	1	2	2
775	PHOSPHATE ION	1	1	1	1	1	1	1	1	1	1	1	1	1	1	1	1
776	PILOCARPINE	1	2	1	1	1	1	1	1	1	1	1	1	1	1	1	1

No.	Name	AR	ARan.	ERα	ERαan.	ERβ	ERβan.	GR	GRan.	LXRα	LXRβ	PPARα	PPARβ	PPARγ	RXRα	TRα	TRβ
777	PIMAVANSERIN	1	2	1	2	1	3	3	2	2	2	1	1	1	1	1	2
778	PIMOZIDE	1	2	4	3	1	4	3	4	4	4	4	4	4	1	2	2
779	PINDOLOL	1	1	1	1	1	1	1	1	1	1	1	1	1	1	2	1
780	PIOGLITAZONE	2	2	1	1	3	1	2	1	2	1	1	1	1	1	2	2
781	PIPERACILLIN	1	1	1	1	1	1	1	1	1	1	1	1	1	1	1	1
782	PIPERONYL BUTOXIDE	1	2	1	1	1	1	1	1	1	1	1	1	1	1	2	1
783	PIRFENIDONE	1	2	1	1	1	1	2	1	1	1	1	1	1	1	2	1
784	PIROXICAM	4	4	3	1	2	1	2	1	1	1	1	1	1	1	1	1
785	PITAVASTATIN	1	2	1	1	1	1	2	1	1	1	1	1	1	1	1	1
786	PITOLISANT	1	2	1	1	1	1	2	1	1	1	1	1	1	1	2	2
787	PLAZOMICIN	1	1	1	1	1	1	1	2	1	1	1	1	1	1	1	1
788	PLERIXAFOR	1	1	3	4	1	4	1	3	2	1	1	1	2	1	1	1
789	PODOFILOX	1	2	1	1	1	1	1	2	1	1	1	1	1	1	1	1
790	POMALIDOMIDE	4	3	2	2	2	2	2	1	1	1	1	1	1	1	2	1
791	PONATINIB	1	1	1	2	1	3	2	4	2	1	2	1	4	1	1	1
792	PRALATREXATE	1	1	1	2	1	1	2	1	1	2	1	1	2	1	1	2
793	PRALIDOXIME	1	2	1	1	1	1	1	1	1	1	1	1	1	1	1	1
794	PRAMIPEXOLE	1	2	1	1	1	1	1	1	1	1	1	1	1	1	1	1
795	PRAMOXINE	1	2	1	1	1	1	1	1	1	1	1	1	1	1	2	1
796	PRASTERONE	4	4	4	2	4	4	3	2	2	2	1	1	1	1	2	3
797	PRASUGREL	1	1	1	2	1	1	2	2	1	1	1	1	1	1	2	3
798	PRAVASTATIN	1	2	1	1	1	1	2	1	1	1	1	1	1	1	1	1
799	PRAZIQUANTEL	1	2	1	1	1	3	2	2	1	1	1	1	1	1	2	1
800	PRAZOSIN	1	1	1	1	1	1	2	2	1	1	1	1	1	1	1	1
801	PREDNICARBATE	1	1	1	1	1	1	1	1	1	1	1	1	1	1	1	1
802	PREDNISOLONE	2	2	1	1	2	1	2	1	1	1	1	1	1	1	1	2
803	PREDNISONE	1	2	1	1	1	1	3	1	1	2	1	1	1	1	1	2
804	PREGABALIN	1	1	1	1	1	1	1	1	1	1	1	1	1	1	1	1
805	PRILOCAINE	1	2	1	1	1	1	1	1	1	1	1	1	1	1	1	1

No.	Name	AR	ARan.	ERα	ERαan.	ERβ	ERβan.	GR	GRan.	LXRα	LXRβ	PPARα	PPARβ	PPARγ	RXRα	TRα	TRβ
806	PRIMAQUINE	1	2	1	1	1	1	1	1	1	1	1	1	1	1	2	1
807	PRIMIDONE	1	2	1	1	1	1	1	1	1	1	1	1	1	1	1	1
808	PROBENECID	1	2	1	1	1	1	1	1	1	1	1	1	1	1	2	1
809	PROCAINAMIDE	1	2	1	1	1	1	1	1	1	1	1	1	1	1	1	1
810	PROCARBAZINE	1	2	1	1	1	1	1	1	1	1	1	1	1	1	1	1
811	PROCHLORPERAZINE	1	2	1	1	1	1	2	1	1	1	1	1	1	1	1	1
812	PROGESTERONE	4	4	3	1	1	1	2	1	3	2	1	1	1	1	2	3
813	PROGUANIL	1	1	1	1	1	1	2	1	1	1	1	1	1	1	1	1
814	PROMETHAZINE	1	2	1	1	2	1	2	1	1	1	1	1	1	1	1	1
815	PROPAFENONE	1	1	2	1	1	1	2	1	1	1	1	1	1	1	2	2
816	PROPARACAINE	1	2	1	1	1	1	1	1	1	1	1	1	1	1	2	1
817	PROPOFOL	1	1	1	1	1	1	1	1	1	1	1	1	1	1	1	1
818	PROPRANOLOL	2	2	1	1	1	1	2	1	1	1	1	1	1	1	2	2
819	PROPYLENE GLYCOL	1	2	1	1	1	1	1	1	1	1	1	1	1	1	1	1
820	PROPYLHEXEDRINE	1	2	1	1	1	1	1	1	1	1	1	1	1	1	1	1
821	PROPYLTHIOURACIL	1	2	1	1	1	1	1	1	1	1	1	1	1	1	1	1
822	PROTRIPTYLINE	2	3	3	2	2	4	2	1	1	1	1	1	1	1	3	3
823	PRUCALOPRIDE	1	2	1	1	1	1	1	1	1	1	1	1	1	1	1	2
824	PYRANTEL	1	2	1	1	1	1	1	1	1	1	1	1	1	1	1	1
825	PYRAZINAMIDE	1	1	1	1	1	1	1	1	1	1	1	1	1	1	1	1
826	PYRIDOSTIGMINE	1	2	1	1	1	1	1	1	1	1	1	1	1	1	1	1
827	PYRIDOXINE	1	1	1	1	1	1	1	1	1	1	1	1	1	1	1	1
828	PYRILAMINE	1	2	1	1	1	1	1	1	1	1	1	1	1	1	2	1
829	PYRIMETHAMINE	2	3	1	1	1	1	2	1	1	1	1	1	1	1	2	2
830	PYRITHIONE	1	2	1	1	1	1	1	1	1	1	1	1	1	1	1	1
831	QUAZEPAM	1	2	3	2	2	2	2	2	2	1	1	1	1	1	2	2
832	QUETIAPINE	1	1	1	1	1	1	2	2	1	1	1	1	1	1	2	2
833	QUINAPRILAT	1	3	1	2	1	2	3	2	1	1	1	2	1	1	2	2
834	QUINIDINE	3	2	2	1	1	1	2	1	1	1	1	1	1	1	2	2

No.	Name	AR	ARan.	ERα	ERαan.	ERβ	ERβan.	GR	GRan.	LXRα	LXRβ	PPARα	PPARβ	PPARγ	RXRα	TRα	TRβ
835	QUININE	1	2	1	1	1	1	2	1	1	1	1	1	1	1	1	2
836	RABEPRAZOLE	1	2	1	1	1	1	2	1	1	1	1	1	1	1	2	2
837	RACEPHEDRINE	1	2	1	1	1	1	1	1	1	1	1	1	1	1	1	1
838	RACEPINEPHRINE	1	2	1	1	1	1	1	1	1	1	1	1	1	1	1	1
839	RALOXIFENE	1	1	2	3	1	4	2	2	2	2	2	3	3	1	1	1
840	RALTEGRAVIR	1	1	1	1	1	1	3	2	1	2	1	2	2	1	1	1
841	RAMELTEON	3	3	1	1	1	1	2	1	1	1	1	1	1	1	3	2
842	RAMIPRILAT	1	1	1	1	1	1	2	2	1	1	1	1	1	1	2	2
843	RANITIDINE	1	1	1	1	1	1	1	1	1	1	1	1	1	1	1	1
844	RANOLAZINE	1	1	1	1	1	1	2	2	1	1	1	1	1	1	1	1
845	RASAGILINE	1	1	1	1	1	1	1	1	1	1	1	1	1	1	1	1
846	REGADENOSON	1	2	1	1	1	1	2	1	1	1	1	1	1	1	1	1
847	REGORAFENIB	1	2	4	3	1	4	3	3	3	3	2	4	2	1	1	1
848	REMIFENTANIL	1	1	1	1	1	1	2	1	1	1	1	1	1	1	1	1
849	REPAGLINIDE	1	1	1	2	1	1	2	2	2	1	1	1	1	1	1	1
850	RESORCINOL	1	2	1	1	1	1	1	1	1	1	1	1	1	1	1	1
851	RESORCINOL MONOACETATE	1	2	1	1	1	1	1	1	1	1	1	1	1	1	1	1
852	RETAPAMULIN	1	1	1	1	1	1	1	2	1	1	1	1	1	1	1	1
853	REVEFENACIN	1	1	3	3	1	4	2	4	3	1	4	2	4	1	1	1
854	RIBAVIRIN	1	1	1	1	1	1	1	1	1	1	1	1	1	1	1	1
855	RIBOCICLIB	1	1	1	1	1	1	2	1	1	1	1	1	1	1	1	1
856	RIBOFLAVIN	1	2	1	1	1	1	2	1	1	1	1	1	2	1	1	1
857	RILPIVIRINE	1	1	1	1	1	1	2	1	2	1	1	1	2	1	1	2
858	RILUZOLE	2	2	1	1	1	1	1	1	1	1	1	1	1	1	2	1
859	RIMANTADINE	1	2	1	1	1	1	1	1	1	1	1	1	1	1	1	1
860	RIOCIGUAT	1	1	1	1	1	3	2	2	2	2	1	1	1	1	1	1
861	RISEDRONIC ACID	1	1	1	1	1	1	1	1	1	1	1	1	1	1	1	1
862	RISPERIDONE	1	1	2	4	1	4	3	2	3	4	3	4	3	1	1	1
863	RIVAROXABAN	1	1	2	3	1	2	3	2	1	1	1	1	1	2	2	2

No.	Name	AR	ARan.	ERα	ERαan.	ERβ	ERβan.	GR	GRan.	LXRα	LXRβ	PPARα	PPARβ	PPARγ	RXRα	TRα	TRβ
864	RIVASTIGMINE	1	1	1	1	1	1	1	1	1	1	1	1	1	1	2	1
865	RIZATRIPTAN	1	1	1	1	1	1	2	1	1	1	1	1	1	1	2	2
866	ROCURONIUM	1	1	1	1	1	1	1	3	1	1	1	1	1	1	1	1
867	ROFLUMILAST	1	2	2	1	1	1	2	1	1	1	1	1	1	1	2	1
868	ROLAPITANT	1	1	1	2	1	4	2	2	2	3	1	4	4	1	1	4
869	ROPINIROLE	1	1	1	1	1	1	1	1	1	1	1	1	1	1	2	1
870	ROPIVACAINE	1	3	1	1	1	1	2	1	1	1	1	1	1	1	2	2
871	ROSIGLITAZONE	3	3	1	1	1	1	2	1	1	1	1	1	1	1	2	2
872	ROSUVASTATIN	1	1	1	1	1	1	2	1	1	1	1	1	1	1	1	1
873	ROTIGOTINE	1	2	2	1	1	1	2	1	1	1	1	1	1	1	2	2
874	RUCAPARIB	1	2	1	2	1	3	2	2	2	2	1	1	2	1	2	2
875	RUFINAMIDE	3	2	1	1	1	1	2	1	1	1	1	1	1	1	2	2
876	SACUBITRILAT	1	2	1	2	1	3	2	1	2	1	1	1	2	1	2	2
877	SAFINAMIDE	1	3	1	1	2	1	2	1	1	1	1	1	1	1	3	3
878	SALICYLIC ACID	1	1	1	1	1	1	1	1	1	1	1	1	1	1	1	1
879	SALMETEROL	1	1	1	1	1	1	2	2	1	1	1	1	1	1	2	2
880	SAPROPTERIN	1	1	1	1	1	1	1	1	1	1	1	1	1	1	1	1
881	SARECYCLINE	1	1	1	1	1	1	2	1	1	1	1	1	1	1	1	1
882	SAXAGLIPTIN	2	2	1	1	1	1	2	1	1	1	1	1	1	1	1	2
883	SCOPOLAMINE	1	3	1	1	1	1	2	1	1	1	1	1	1	1	2	2
884	SECNIDAZOLE	1	2	1	1	1	1	1	1	1	1	1	1	1	1	1	1
885	SECOBARBITAL	1	2	1	1	1	1	1	1	1	1	1	1	1	1	1	1
886	SEGESTERONE	4	4	3	3	1	1	3	2	2	2	1	1	1	1	1	4
887	SELEGILINE	1	1	1	1	1	1	1	1	1	1	1	1	1	1	1	1
888	SELENIUM SULFIDE	1	1	1	1	1	1	1	1	1	1	1	1	1	1	1	1
889	SELEXIPAG	1	1	1	1	1	1	2	2	1	1	1	1	2	1	1	1
890	SERTACONAZOLE	1	2	3	1	1	1	2	2	2	1	1	1	2	1	1	1
891	SERTRALINE	1	1	4	2	3	2	2	2	1	1	1	1	1	1	2	2
892	SEVOFLURANE	1	2	1	1	1	1	1	1	1	1	1	1	1	1	1	1

No.	Name	AR	ARan.	ERα	ERαan.	ERβ	ERβan.	GR	GRan.	LXRα	LXRβ	PPARα	PPARβ	PPARγ	RXRα	TRα	TRβ
893	SILDENAFIL	1	1	1	1	1	1	2	1	1	1	1	1	2	1	1	1
894	SILODOSIN	1	2	1	1	1	1	2	1	1	1	2	1	2	1	1	1
895	SIMVASTATIN	1	1	1	1	1	1	2	2	1	1	1	1	1	1	1	1
896	SITAGLIPTIN	3	3	4	1	2	2	2	2	2	2	1	1	2	2	2	3
897	SOFOSBUVIR	1	2	1	1	1	1	2	2	1	1	2	1	1	1	1	1
898	SOLIFENACIN	1	3	1	2	1	4	2	3	1	2	1	1	1	1	1	2
899	SONIDEGIB	1	2	1	3	1	4	2	3	2	2	1	1	1	2	1	1
900	SORAFENIB	1	1	2	4	1	2	2	2	3	2	1	1	2	1	1	1
901	SORBITOL	1	2	1	1	1	1	1	1	1	1	1	1	1	1	1	1
902	SOTALOL	1	1	1	1	1	1	1	1	1	1	1	1	1	1	2	1
903	SPIRONOLACTONE	1	2	1	1	1	1	1	2	1	3	1	1	1	1	1	1
904	STAVUDINE	1	1	1	1	1	1	1	1	1	1	1	1	1	1	1	1
905	STIRIPENTOL	2	2	1	1	1	1	2	1	1	1	1	1	1	1	2	2
906	STREPTOMYCIN	1	1	1	1	1	1	2	1	1	1	1	1	1	1	1	1
907	STREPTOZOCIN	1	2	1	1	1	1	1	1	1	1	1	1	1	1	1	1
908	SUCCINYLCHOLINE	1	2	1	1	1	1	1	1	1	1	1	1	1	1	1	1
909	SUFENTANIL	1	1	1	1	1	1	2	2	1	1	1	1	1	1	1	1
910	SULBACTAM	1	2	1	1	1	1	1	1	1	1	1	1	1	1	1	1
911	SULCONAZOLE	1	2	1	1	1	2	2	1	1	1	1	1	1	1	2	2
912	SULFACETAMIDE	1	2	1	1	1	1	1	1	1	1	1	1	1	1	1	1
913	SULFADIAZINE	1	3	1	1	1	1	1	1	1	1	1	1	1	1	2	1
914	SULFAMETHOXAZOLE	1	2	1	1	1	1	1	1	1	1	1	1	1	1	2	1
915	SULFANILAMIDE	1	2	1	1	1	1	1	1	1	1	1	1	1	1	1	1
916	SULFASALAZINE	1	1	1	2	1	1	2	2	2	1	1	1	2	1	2	2
917	SULFATE ION	1	1	1	1	1	1	1	1	1	1	1	1	1	1	1	1
918	SULFUR	1	2	1	1	1	1	1	1	1	1	1	1	1	1	1	1
919	SULINDAC	1	1	1	1	1	2	2	1	2	1	1	1	1	1	1	3
920	SUMATRIPTAN	1	1	1	1	1	1	1	1	1	1	1	1	1	1	2	1
921	SUNITINIB	1	1	1	1	1	1	2	2	2	2	1	1	1	1	2	2

No.	Name	AR	ARan.	ERα	ERαan.	ERβ	ERβan.	GR	GRan.	LXRα	LXRβ	PPARα	PPARβ	PPARγ	RXRα	TRα	TRβ
922	SUVOREXANT	1	1	1	1	1	1	1	1	1	1	1	1	2	1	1	1
923	TADALAFIL	1	1	3	4	1	4	1	3	1	1	1	3	3	1	1	1
924	TAFAMIDIS	2	2	1	1	1	1	2	1	1	1	1	1	1	1	2	2
925	TAFENOQUINE	1	2	1	1	1	1	2	1	1	1	1	1	1	1	1	1
926	TAFLUPROST	1	2	2	1	1	1	2	1	1	1	1	1	1	1	1	2
927	TALAZOPARIB	1	1	2	1	1	1	2	3	1	2	1	2	2	1	1	1
928	TAMOXIFEN	1	2	4	3	1	4	2	1	1	1	1	1	1	1	1	1
929	TAMSULOSIN	1	1	1	1	1	1	2	1	1	1	1	1	1	1	1	1
930	TAPENTADOL	1	2	1	1	1	1	2	1	1	1	1	1	1	1	2	1
931	TASIMELTEON	2	3	1	1	2	1	2	1	1	1	1	1	1	1	2	2
932	TAZAROTENE	1	1	1	1	1	1	2	1	1	2	1	1	1	1	1	2
933	TAZOBACTAM	1	2	1	1	1	1	1	1	1	1	1	1	1	1	1	1
934	TECOVIRIMAT	1	3	2	1	1	2	2	1	2	2	1	2	1	2	1	2
935	TEDIZOLID	1	1	1	1	1	1	3	1	2	2	1	1	1	1	3	3
936	TELMISARTAN	1	1	1	4	1	4	1	3	3	2	3	2	4	1	1	1
937	TELOTRISTAT	1	1	1	2	1	1	3	2	3	1	4	1	4	1	1	1
938	TEMAZEPAM	1	2	4	1	2	3	2	1	1	1	1	1	1	1	1	1
939	TEMOZOLOMIDE	1	1	1	1	1	1	1	1	1	1	1	1	1	1	1	1
940	TERAZOSIN	1	1	1	1	1	1	2	2	1	1	1	1	1	1	1	1
941	TERBINAFINE	1	1	2	2	1	2	2	2	2	1	1	1	1	1	2	2
942	TERBUTALINE	1	2	1	1	1	1	1	1	1	1	1	1	1	1	1	1
943	TERCONAZOLE	1	1	3	3	1	3	2	3	3	2	3	1	1	1	1	1
944	TERIFLUNOMIDE	2	3	1	1	1	1	2	1	1	1	1	1	1	1	3	2
945	TESTOSTERONE	4	4	3	3	3	3	2	1	2	2	1	1	1	1	2	2
946	TETRABENAZINE	1	2	1	1	1	1	2	1	1	1	1	1	1	1	1	1
947	TETRACAINE	1	2	1	1	1	1	1	1	1	1	1	1	1	1	2	1
948	TETRACYCLINE	1	1	1	1	1	1	1	1	1	1	1	1	1	1	1	1
949	TETRADECYL HYDROGEN SULFATE (ESTER)	1	2	1	1	1	1	1	1	1	1	1	1	1	1	1	1
950	TETRAHYDROZOLINE	2	3	1	1	1	1	2	1	1	1	1	1	1	1	2	2

No.	Name	AR	ARan.	ERα	ERαan.	ERβ	ERβan.	GR	GRan.	LXRα	LXRβ	PPARα	PPARβ	PPARγ	RXRα	TRα	TRβ
951	TEZACAFTOR	1	1	1	1	1	1	2	2	2	1	1	1	1	1	1	1
952	THALIDOMIDE	4	3	2	2	2	1	2	2	1	1	1	1	1	1	2	2
953	THEOPHYLLINE	1	1	1	1	1	1	1	1	1	1	1	1	1	1	1	1
954	THIAMINE ION	1	2	1	1	1	1	1	1	1	1	1	1	1	1	1	1
955	THIOGUANINE	1	2	1	1	1	1	1	1	1	1	1	1	1	1	1	1
956	THIORIDAZINE	1	1	1	1	1	1	1	1	1	1	1	1	1	1	1	1
957	THIOSULFATE ION	1	1	1	1	1	1	1	1	1	1	1	1	1	1	1	1
958	THIOTEPA	1	2	1	1	1	1	1	1	1	1	1	1	1	1	1	1
959	THIOTHIXENE	1	1	1	1	1	1	2	1	1	1	1	1	2	1	1	1
960	THONZONIUM	1	2	1	1	1	1	2	1	1	1	1	1	1	1	1	1
961	THONZYLAMINE	1	2	1	1	1	1	1	1	1	1	1	1	1	1	1	1
962	TIAGABINE	1	2	2	1	1	1	2	1	1	1	1	1	1	1	2	1
963	TICAGRELOR	1	1	1	1	1	1	2	2	1	1	1	1	1	1	1	1
964	TICLOPIDINE	2	2	1	1	1	1	2	1	1	1	1	1	1	1	2	2
965	TIGECYCLINE	1	1	1	3	1	1	1	3	1	1	1	2	1	1	1	1
966	TIMOLOL	1	1	1	1	1	1	1	1	1	1	1	1	1	1	1	1
967	TINIDAZOLE	1	2	1	1	1	1	1	1	1	1	1	1	1	1	1	1
968	TIOCONAZOLE	1	2	1	1	1	1	2	1	1	1	1	1	1	1	1	1
969	TIOPRONIN	1	2	1	1	1	1	1	1	1	1	1	1	1	1	1	1
970	TIOTROPIUM	1	1	1	1	1	1	2	2	1	1	1	1	1	1	1	1
971	TIPIRACIL	1	3	1	1	1	1	1	1	1	1	1	1	1	1	1	1
972	TIZANIDINE	1	1	1	1	1	1	1	1	1	1	1	1	1	1	2	1
973	TIZOXANIDE	3	2	1	1	1	1	1	1	1	1	1	1	1	1	2	2
974	TOBRAMYCIN	1	1	1	1	1	1	1	1	1	1	1	1	1	1	1	1
975	TOFACITINIB	1	3	1	1	1	1	2	1	1	1	1	1	1	1	1	1
976	TOLAZAMIDE	1	2	1	1	2	1	2	1	1	1	1	1	1	1	2	2
977	TOLBUTAMIDE	1	2	1	1	1	1	1	1	1	1	1	1	1	1	2	1
978	TOLCAPONE	2	3	2	2	2	1	2	2	1	1	2	1	1	1	3	2
979	TOLMETIN	1	2	1	1	1	1	2	1	1	1	1	1	1	1	2	2

No.	Name	AR	ARan.	ERα	ERαan.	ERβ	ERβan.	GR	GRan.	LXRα	LXRβ	PPARα	PPARβ	PPARγ	RXRα	TRα	TRβ
980	TOLNAFTATE	1	3	4	2	3	3	2	2	2	2	1	2	2	1	3	3
981	TOLTERODINE	1	2	2	3	2	2	2	2	1	1	1	1	1	1	2	2
982	TOLVAPTAN	1	1	1	1	1	4	2	4	1	1	3	4	3	1	1	1
983	TOPIRAMATE	1	1	1	1	1	1	2	2	1	1	1	1	1	1	1	1
984	TOPOTECAN	1	1	1	1	1	1	2	2	1	3	1	1	2	1	1	1
985	TOREMIFENE	1	2	4	3	1	3	2	1	1	1	1	1	1	1	1	1
986	TORSEMIDE	1	1	1	2	1	1	2	2	1	1	1	1	1	1	2	2
987	TRAMADOL	1	1	1	1	1	1	1	1	1	1	1	1	1	1	1	1
988	TRANDOLAPRILAT	1	1	2	1	1	1	2	2	1	1	1	1	1	2	2	1
989	TRANEXAMIC ACID	1	2	1	1	1	1	1	1	1	1	1	1	1	1	1	1
990	TRAVOPROST	1	1	2	3	1	2	2	2	1	1	1	1	1	1	1	1
991	TRAZODONE	1	1	1	2	1	1	2	2	1	1	1	2	2	1	2	2
992	TREPROSTINIL	1	1	1	1	1	1	2	1	1	1	1	1	1	1	2	2
993	TRETINOIN	1	1	1	1	1	1	2	1	2	1	1	1	1	1	3	3
994	TRIAMCINOLONE ACETONIDE	1	1	1	1	1	1	2	2	1	2	1	1	1	1	1	1
995	TRIAMTERENE	4	4	3	2	2	2	2	1	2	1	1	1	2	1	3	2
996	TRIAZOLAM	1	2	3	2	1	4	2	3	1	1	1	1	1	1	1	1
997	TRICLABENDAZOLE	1	2	1	1	1	1	2	1	1	1	1	1	1	1	3	2
998	TRIENTINE	1	2	1	1	1	1	1	1	1	1	1	1	1	1	1	1
999	TRIFLUOPERAZINE	1	2	1	2	1	2	2	2	1	1	1	1	1	1	1	1
1000	TRIFLURIDINE	2	3	2	1	2	1	2	1	1	1	1	1	1	1	2	2
1001	TRIHEXYPHENIDYL	3	4	3	2	4	3	2	1	1	1	1	1	1	1	2	2
1002	TRIMETHOBENZAMIDE	1	2	1	1	1	1	2	1	1	1	1	1	1	1	1	1
1003	TRIMETHOPRIM	1	2	1	1	1	1	1	1	1	1	1	1	1	1	2	2
1004	TRIMIPRAMINE	1	3	3	2	2	3	2	1	1	1	1	1	1	1	1	2
1005	TRIPROLIDINE	1	3	3	2	1	3	1	1	1	1	1	1	1	1	1	2
1006	TROPICAMIDE	1	1	1	1	1	1	2	1	1	1	1	1	1	1	2	2
1007	TROSPIUM	1	1	1	1	1	2	2	2	3	2	1	1	1	1	1	2
1008	ULIPRISTAL	1	1	3	4	1	1	1	1	1	1	1	1	1	1	1	1

No.	Name	AR	ARan.	ERα	ERαan.	ERβ	ERβan.	GR	GRan.	LXRα	LXRβ	PPARα	PPARβ	PPARγ	RXRα	TRα	TRβ
1009	UMECLIDINIUM	1	2	4	2	1	3	3	3	1	2	1	1	2	1	1	1
1010	UNDECYLENIC ACID	1	2	1	1	1	1	1	1	1	1	1	1	1	1	1	1
1011	UREA	1	2	1	1	1	1	1	1	1	1	1	1	1	1	1	1
1012	UREA C-13	1	2	1	1	1	1	1	1	1	1	1	1	1	1	1	1
1013	UREA C-14	1	2	1	1	1	1	1	1	1	1	1	1	1	1	1	1
1014	URIDINE	1	2	1	1	1	1	1	1	1	1	1	1	1	1	1	1
1015	URSODIOL	1	2	1	1	1	1	2	1	1	2	1	1	1	1	1	2
1016	VALPROIC ACID	1	1	1	1	1	1	1	1	1	1	1	1	1	1	1	1
1017	VALSARTAN	1	1	1	3	1	2	2	2	1	2	1	1	1	1	1	1
1018	VANDETANIB	1	2	1	1	1	1	2	1	2	1	2	1	2	1	2	2
1019	VARDENAFIL	1	1	1	1	1	1	1	2	1	1	1	1	2	1	1	1
1020	VARENICLINE	2	2	1	1	1	1	1	1	1	1	1	1	1	1	2	2
1021	VECURONIUM	1	1	1	1	1	1	1	2	1	1	1	1	1	1	1	1
1022	VEMURAFENIB	1	1	1	1	1	3	2	3	3	2	2	2	1	3	1	1
1023	VENLAFAXINE	1	1	1	1	1	1	1	1	1	1	1	1	1	1	2	1
1024	VERAPAMIL	1	1	1	1	1	1	2	2	1	1	1	1	1	1	1	1
1025	VIDARABINE	2	2	1	1	1	1	1	1	1	1	1	1	1	1	1	1
1026	VIGABATRIN	1	1	1	1	1	1	1	1	1	1	1	1	1	1	1	1
1027	VILANTEROL	1	2	1	1	1	1	2	1	1	1	1	1	1	1	2	1
1028	VILAZODONE	1	1	1	2	1	1	4	3	2	2	2	3	2	1	1	2
1029	VISMODEGIB	1	2	1	1	1	1	3	2	2	2	1	1	1	2	1	2
1030	VORAPAXAR	1	1	1	3	1	4	1	4	1	1	2	1	2	1	1	1
1031	VORICONAZOLE	2	4	2	2	2	2	2	2	1	1	1	1	1	1	2	1
1032	VORINOSTAT	1	2	1	1	1	1	1	1	1	1	1	1	1	1	2	2
1033	VORTIOXETINE	1	4	2	1	2	1	2	1	1	1	1	1	1	1	2	2
1034	WARFARIN	1	3	2	4	2	3	2	2	1	1	1	1	1	1	3	3
1035	XYLOMETAZOLINE	1	2	1	1	1	1	2	1	1	1	1	1	1	1	2	2
1036	ZAFIRLUKAST	1	1	1	1	1	4	2	3	3	1	2	1	4	1	1	1
1037	ZALEPLON	1	2	1	1	1	1	2	1	1	1	1	1	1	1	2	2

No.	Name	AR	ARan.	ERα	ERαan.	ERβ	ERβan.	GR	GRan.	LXRα	LXRβ	PPARα	PPARβ	PPARγ	RXRα	TRα	TRβ
1038	ZANAMIVIR	1	1	1	1	1	1	1	1	1	1	1	1	1	1	1	1
1039	ZIDOVUDINE	2	3	1	1	1	1	2	1	1	1	1	1	1	1	1	1
1040	ZILEUTON	2	2	1	1	1	1	2	1	1	1	1	1	1	1	2	1
1041	ZINC CATION	1	1	1	1	1	1	1	1	1	1	1	1	1	1	1	1
1042	ZIPRASIDONE	1	1	2	3	1	2	3	2	3	2	1	1	2	1	1	3
1043	ZOLEDRONIC ACID	1	1	1	1	1	1	1	1	1	1	1	1	1	1	1	1
1044	ZOLMITRIPTAN	2	1	2	1	1	2	2	2	1	1	1	1	1	1	2	2
1045	ZOLPIDEM	1	1	1	2	1	1	2	2	2	1	1	1	1	1	2	2
1046	ZONISAMIDE	1	1	1	1	1	1	1	1	1	1	1	1	1	1	1	1

[1] 1 – class green in ED corresponds to low probability of binding; 2 and 3 – class yellow and orange, respectively, correspond to medium probability of binding; 4 – class red corresponds to high probability of binding; AR – agonist conformation of androgen receptor; ARan. – antagonist conformation of androgen receptor; ERα – agonist conformation of estrogen receptor α; ERαan. – antagonist conformation of estrogen receptor α; ERβ – agonist conformation of estrogen receptor β; ERβ – antagonist conformation of estrogen receptor β; GR – agonist conformation of glucocorticoid receptor; GR – antagonist conformation of glucocorticoid receptor; LXRα – liver X receptor α; LXRβ – liver X receptor β; PPARα – peroxisome proliferator activated receptor α; PPARβ – peroxisome proliferator activated receptor β; PPARγ – peroxisome proliferator activated receptor γ; RXRα – retinoid X receptor α ; TRα – thyroid receptor α; TRβ – thyroid receptor β.
